# Closed–Loop ventilation using sidestream versus mainstream capnography for automated adjustments of minute ventilation—A randomized clinical trial in cardiac surgery patients

**DOI:** 10.1371/journal.pone.0289412

**Published:** 2023-08-23

**Authors:** Sunny G. L. H. Nijbroek, Jan-Paul Roozeman, Sarah Ettayeby, Neeltje M. Rosenberg, David M. P. van Meenen, Thomas G. V. Cherpanath, Wim K. Lagrand, Robert Tepaske, Robert J. M. Klautz, Ary Serpa Neto, Marcus J. Schultz

**Affiliations:** 1 Department of Intensive Care, Amsterdam University Medical Centers, Location AMC, Amsterdam, The Netherlands; 2 Department of Anesthesiology, Amsterdam University Medical Centers, Location AMC, Amsterdam, The Netherlands; 3 Department of Internal Medicine, Spaarne Hospital, Haarlem, The Netherlands; 4 Department of Cardiothoracic Surgery, Amsterdam University Medical Centers, Location AMC, Amsterdam, The Netherlands; 5 Department of Cardiothoracic Surgery, Leiden University Medical Center, Leiden, The Netherlands; 6 Australian and New Zealand Intensive Care Research Centre (ANZIC–RC), School of Public Health and Preventive Medicine, Monash University, Melbourne, Australia; 7 Laboratory of Experimental Intensive Care and Anesthesiology (L·E·I·C·A), Amsterdam University Medical Centers, Location AMC, Amsterdam, The Netherlands; 8 Department of Critical Care, Austin Hospital, Melbourne Medical School, University of Melbourne, Melbourne, Australia; 9 Department of Critical Care Medicine, Hospital Israelita Albert Einstein, Sao Paolo, Brazil; 10 Nuffield Department of Medicine, University of Oxford, Oxford, United Kingdom; 11 Mahidol–Oxford Tropical Medicine Research Unit (MORU), Mahidol University, Bangkok, Thailand; Sant Anna Hospital: Clinica Sant’Anna, SWITZERLAND

## Abstract

**Background:**

INTELLiVENT–Adaptive Support Ventilation (ASV) is a closed–loop ventilation mode that uses capnography to adjust tidal volume (V_T_) and respiratory rate according to a user–set end–tidal CO_2_ (etCO_2_) target range. We compared sidestream versus mainstream capnography with this ventilation mode with respect to the quality of breathing in patients after cardiac surgery.

**Methods:**

Single–center, single–blinded, non–inferiority, randomized clinical trial in adult patients scheduled for elective cardiac surgery that were expected to receive at least two hours of postoperative ventilation in the ICU. Patients were randomized 1:1 to closed–loop ventilation with sidestream or mainstream capnography. Each breath was classified into a zone based on the measured V_T_, maximum airway pressure, etCO_2_ and pulse oximetry. The primary outcome was the proportion of breaths spent in a predefined ‘optimal’ zone of ventilation during the first three hours of postoperative ventilation, with a non–inferiority margin for the difference in the proportions set at –20%. Secondary endpoints included the proportion of breaths in predefined ‘acceptable’ and ‘critical’ zones of ventilation, and the proportion of breaths with hypoxemia.

**Results:**

Of 80 randomized subjects, 78 were included in the intention–to–treat analysis. We could not confirm the non–inferiority of closed–loop ventilation using sidestream with respect to the proportion of breaths in the ‘optimal’ zone (mean ratio 0.87 [0.77 to ∞]; *P* = 0.116 for non–inferiority). The proportion of breaths with hypoxemia was higher in the sidestream capnography group versus the mainstream capnography group.

**Conclusions:**

We could not confirm that INTELLiVENT–ASV using sidestream capnography is non–inferior to INTELLiVENT–ASV using mainstream capnography with respect to the quality of breathing in subjects receiving postoperative ventilation after cardiac surgery.

**Trial registration:**

NCT04599491 (clinicaltrials.gov).

## Introduction

INTELLiVENT–adaptive support ventilation (ASV) is a closed–loop ventilation mode that uses pressure–controlled or pressure support ventilation depending on patients’ activity. Continuous pulse oximetry and capnography are used for adjustments of positive end–expiratory pressure (PEEP) and fraction of inspired oxygen (FiO_2_), and minute volume, respectively. Automatic adjustments are performed based on two equations, named ‘Otis’ and ‘Mead’ in order to reach the lowest work of breathing and force of breathing, respectively, according to user–set target ranges for end–tidal carbon dioxide (etCO_2_). Herein, the ventilator titrates the inspiratory pressure to reach an ‘optimal’ combination of tidal volume (V_T_) and respiratory rate (RR) [1–3]. This mode has been shown to be safe and effective with respect to the quality of breathing in various types of critically ill patients [4–6], and also in patients receiving ventilation after cardiac surgery [7–9].

With INTELLiVENT–ASV, either sidestream or mainstream capnography can be used, although these techniques have never been compared directly [10]. Sidestream capnography is mostly used during ventilation in the operating room, and it can be used in spontaneously breathing patients as well as pediatric patients where added weight and dead space in the airway circuit is undesirable [11–13]. The sidestream capnography technique causes less weight on the airway and the sensor is less fragile. One disadvantage of sidestream capnography, however, is the slight delay in readings due to sampling and transport of the air mixture to the sensor. Other disadvantages include poor readings due to accidental crushing or kinking of the sampling tube, and potential blockages by condensation from humidified sample gas, and airway secretions [14, 15]. In the adult ICU setting, mainstream capnography is most often used and widely available [16]. Mainstream capnography has the disadvantage that it increases dead space, and adds additional weight to the artificial airway, caused by the sensor block that is usually placed close to the distal end of the endotracheal tube [15]. Additionally, this technique is costlier than sidestream capnography, and the sensor could easily be damaged during cleaning and storage [14, 15].

Thus far, clinical studies of INTELLiVENT–ASV only tested its safety and efficacy when using mainstream capnography. Sidestream capnography could serve as an attractive alternative for mainstream capnography, in certain settings and in several patient categories. The differences in the accuracies of the two techniques, however, could affect the performance of the closed–loop ventilation mode [17, 18]. Therefore, we performed a head–to–head comparison of these two alternate ways of capnography in a randomized clinical trial named ‘INTELLiVENT–ASV using mainstream versus sidestream capnography in cardiac surgery patients’ (INTELLiSTREAM). We hypothesized that closed–loop ventilation using sidestream capnography is non–inferior to closed–loop ventilation using mainstream capnography with respect to the quality of breathing.

## Methods

### Study design and oversight

INTELLiSTREAM was an investigator–initiated and investigator–sponsored, single-center, single–blinded, non–inferiority, randomized clinical trial comparing postoperative closed–loop ventilation using sidestream versus mainstream capnography. The study was performed at the Amsterdam University Medical Centers, location ‘AMC’, Amsterdam, the Netherlands. The trial was approved by the local Institutional Review Board (METC 2019_222; Chair prof. dr. J.A. Swinkels; date of ethical approval march 6^th^, 2020) and registered at clinicaltrials.gov (study identifier NCT04599491). INTELLiSTREAM was performed in accordance to the principles of the Declaration of Helsinki, adhering to Good Clinical Practice guidelines including data monitoring. Written informed consent was obtained from all subjects prior to surgery.

### Study population

Subjects were eligible for participation if: (1) aged ≥ 18 years; (2) scheduled for elective cardiac surgery; and (3) expected to require at least two hours of ventilation after arrival in the ICU, e.g., patients scheduled for open valve surgery, aortic surgery or on–pump coronary artery bypass grafting. Exclusion criteria were previous inclusion in this study, and participation in another intervention that could influence ventilator settings and ventilation parameters. We also excluded subjects with confirmed or suspected pregnancy, and subjects that were expected to die in the first hours after surgery.

### Randomization

Subjects were randomized in a 1:1 ratio to receive closed–loop ventilation using sidestream or mainstream capnography. Randomization was performed by one of the study coordinators at the end of the surgical procedure shortly before transport to the ICU for postoperative care, using a web–based, password–protected program (http://www.castoredc.com), and permuted blocks with random block sizes. Subjects remained blinded to which capnography sensor was used during postoperative ventilation. Healthcare professionals could not be blinded because of the nature of the intervention. The investigator performing the statistical analysis, however, remained blinded for treatment allocation when analyzing the primary endpoint.

### Study procedures and data collection

In the ICU, subjects were typically cared for by one dedicated board–certified ICU nurse. Changes in treatment were implemented based on observations by the nurse, and according to the recommendations in the local guideline for postoperative care. Closed-loop ventilation was generally initiated within 10 minutes of ICU arrival, with automated adjustments of minute volume, PEEP and FiO_2_ activated. Data was collected breath-by-breath until tracheal extubation, or up to a maximum of 6 hours, using a Memorybox data storage device (Hamilton Medical AG) attached directly to the ventilator. Extubation was performed according to criteria described in the local protocol. Follow-up was performed until 30 days after randomization. Further details regarding data collection and routine perioperative care can be found in the S1 File. This study was performed in accordance with CONSORT guidelines for randomized controlled trials.

### Endpoints

Quality of breathing was scored using three predefined and previously used ventilation zones, named ‘optimal’, ‘acceptable’, and critical’ (**Table 1**) [9]. For this, each breath was classified into one of these three zone based on the measured V_T_, maximum airway pressure (Pmax), etCO_2_ and peripheral oxygen saturation by pulse oximetry (SpO_2_). We chose to use these definitions, to make the study results comparable to previous [9] and future studies on automated ventilation [19]. The primary endpoint was the *proportion of breaths* spent in the ‘optimal zone´ of ventilation during the first 3 hours of postoperative ventilation. In case of missing data, if all of the parameters were missing, the zone was considered missing. If parameters were missing, but one was available and in the critical zone, the zone was defined as critical. If parameters were missing, but one was available and not in the critical zone, the zone was defined as missing.

**Table 1:** The predefined ventilation zones*.

	Critical Zone	Acceptable Zone	Optimal Zone
**Tidal volume, ml/kg PBW**	> 12	8–12	≤ 8
	***OR***	***AND/OR***	***AND***
Maximum airway pressure, cm H_2_O	≥ 36	31–36	≤ 30
	***OR***	***AND/OR***	***AND***
etCO_2_, mmHg	< 25 OR ≥ 51	25–30 OR 46–51	30–46
	***OR***	***AND/OR***	***AND***
SpO_2_, %	< 85	≥ 98 *OR* 85–93	93–98 *OR* ≥ 93 if FiO_2_ ≤ 40%
**Definitions**	if any parameters present: *‘critical zone’*	no parameters in *‘critical zone’*, but not all parameters in *‘optimal zone’*: *‘acceptable zone’*	if all parameters present: *‘optimal zone’*

* Zones of ventilation adapted from: De Bie et al. [9]

These definitions have been used in previous studies on automated ventilation and they reflect the quality of breathing with respect to important ventilator variables, using generally accepted safety zones.

Abbreviations: etCO2: end–tidal carbon dioxide by mainstream capnography; FiO2: fraction of inspired oxygen; PBW, predicted body weight; SpO2: oxygen saturation by pulse oximetry

We had the following secondary endpoints: the proportion of breaths spent in the ‘acceptable’ and ‘critical’ zone in the first 3 hours of postoperative ventilation; the *proportion of time* spent in the predefined ventilation zones in the first 3 hours of postoperative ventilation; duration of weaning, defined as the time from cessation of sedatives until tracheal extubation; and total duration of postoperative ventilation, defined as the time from the start of ventilation in the ICU until tracheal extubation. We also collected the following endpoints: the proportion of failed extubations, defined as a reintubation within 48 hours after extubation, excluding subjects for a re–thoracotomy; incidence of postoperative pulmonary complications, defined as a collapsed composite of pneumonia, pneumothorax or severe atelectasis; length of stay in ICU and hospital; ICU readmission; mortality in ICU and at day 30; the incidence of hypoxemia, defined as the proportion of breaths with an SpO_2_ < 85%, but only when the SpO_2_ had a quality index of > 50%, as well as ventilation parameters and the results from clinically–indicated arterial blood gas analyses.

### Sample size calculation

We estimated that a sample size of 72 subjects would provide 80% power, considering an expected proportion of breaths in the ‘optimal’ zone of ventilation with mainstream capnography of 69 ± 23% [9], with a one–sided alpha level of 0.05 and a non–inferiority margin of 20%. This margin corresponds to 14% less breaths in the ‘optimal’ zone in the sidestream group compared to the mainstream group. Subjects that were extubated before 90 minutes of postoperative ventilation, were considered drop–outs and were replaced with new subjects. Thus, enrollment was continued until both study arms had at least 36 subjects with the minimum duration of 90 minutes of postoperative ventilation. The cutoff of 90 minutes was chosen to have sufficient time to observe the evolution of postoperative ventilation requirements, to increase the comparability of subjects, and to reduce the likelihood of clinical and statistical heterogeneity, as subjects rapidly awaking from anesthesia after surgery would likely have different respiratory needs.

### Statistical analysis

For the intention to treat analysis, we analyzed the recorded ventilation data according to the treatment that subjects actually received. Continuous variables are presented as medians with interquartile ranges (IQR), and categorical variables with numbers and proportions. Continuous data are presented as medians (quartile 25% to quartile 75%), regardless of distribution, for consistency and to enhance readability.

The proportion of breaths in the ‘optimal’, ‘acceptable’ and ‘critical’ zones is summarized per subject according to the following equation:

proportionofbreathsinzone=(numberofbreathsinzone/totalnumberofbreaths) (Eq 1)

Data for the primary outcome are presented as median (quartile 25% to quartile 75%) and mean ± standard deviation, and compared as a mean ratio (Eq 2) tested for non–inferiority considering a margin of 20%, and presented with a one–sided 95% confidence interval (95%–CI):

MeanRatio=(MeanproportionofbreathsinzoneforSidestream/MeanproportionofbreathsinzoneforMainstream) (Eq 2)

A one–sided *P* value for non–inferiority was calculated. Non–inferiority was established if the lower boundary of the one–sided 95% confidence interval was higher than 0.80 (indicating 20% decrease in proportion of breaths in the optimal zone). If non–inferiority was confirmed, superiority for sidestream capnography was to be tested considering a 95% CI following hierarchical closed testing procedure.

All analysis for secondary endpoints tested for superiority and were two–sided. For secondary outcomes assessing the proportion of breaths and the incidence of hypoxemia, the denominator was the total number of breaths. The proportion of breaths in acceptable and critical zones, or with hypoxemia were analyzed using the same strategy as the primary outcome. For all other secondary endpoints, effect estimates were calculated using univariate logistic or linear regression, for binary and continuous endpoints respectively.

In creating the per protocol analysis, we excluded subjects who had one or more major protocol violations, i.e., not meeting minimum ventilation time requirement, or receiving ventilation with the capnography sensor that they were not assigned to.

Treatment effect on the primary outcome was analyzed according to following subgroups: 1) subjects who were successfully extubated before or after median postoperative ventilation duration; 2) subjects with shorter or longer than median intraoperative ventilation duration; and 3) subjects with PaO_2_/FiO_2_ ratio below or above the median at admission in the ICU. The effect of subgroups was evaluated according to the interaction effects between each subgroup and the study arms following the primary analysis.

For the analyses of ventilation parameters over the first three hours of ventilation, all parameters were summarized as the mean of every 5 minutes until extubation or 180 minutes, whichever came first. Sidestream and mainstream groups were compared using mixed–effect longitudinal models with the subjects as random effect, the variable of interest as the dependent variable and the time of measurement, randomization group and an interaction of time and randomization group as fixed effects.

The effects of the intervention on the duration of weaning, and the time until ICU and hospital discharge were assessed using Kaplan–Meier survival curves and a log rank test to calculate a two–sided *P* value for hypothesis testing. Survival time was calculated from the time of randomization until the time of the outcome. Patients who died during follow–up were censored for Kaplan–Meier estimates. Lastly, for quality checking we plotted a comparison between sensors of capnography measurements every 10 minutes for the first three hours of postoperative ventilation.

All analyses were performed using R statistical software (version 4.1.0, R Core Team, 2016, Vienna, Austria), and a *P* < 0.05 was considered statistically significant.

## Results

### Cohort characteristics

**Fig 1:**
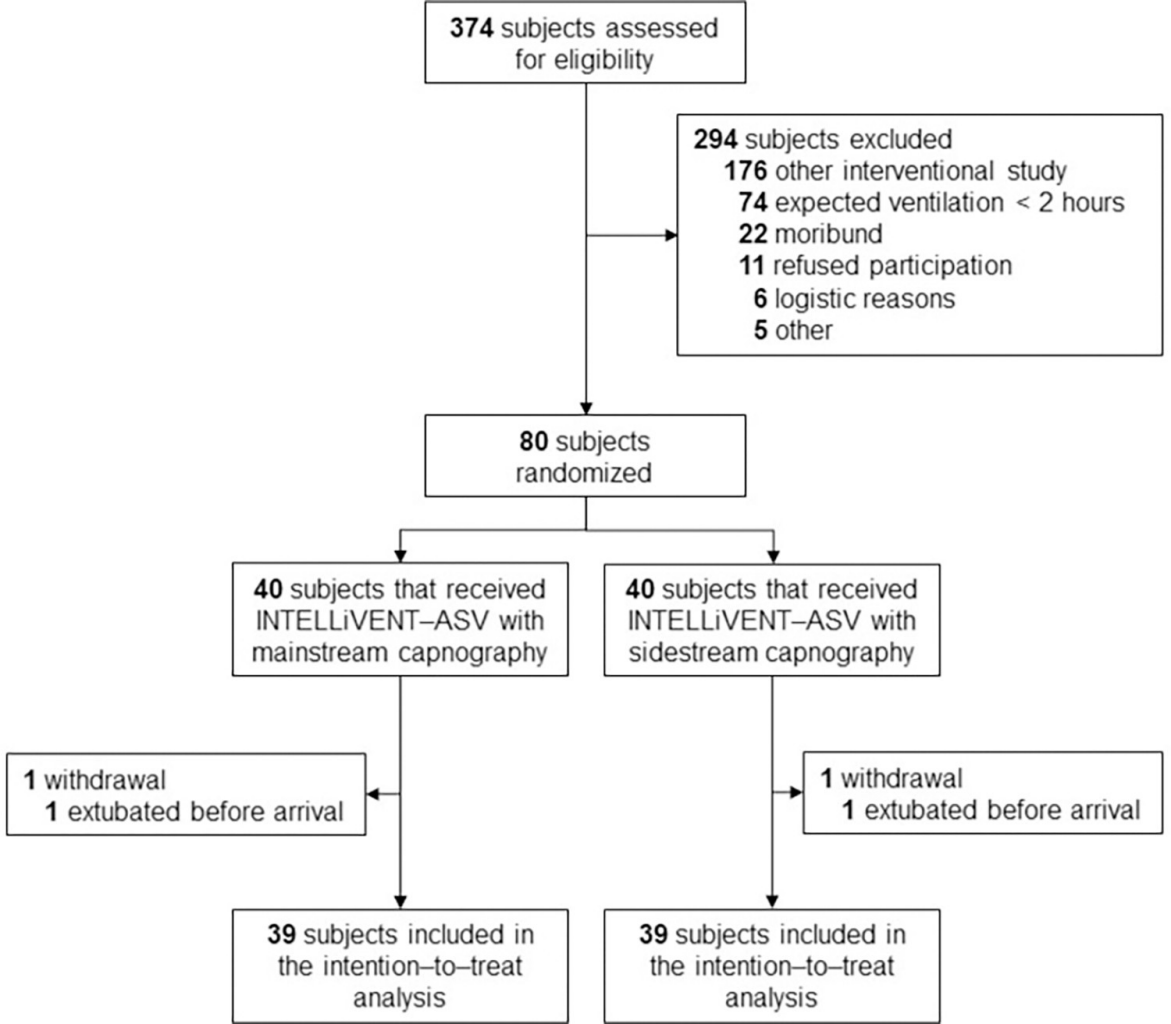
CONSORT flow diagram.

Between June 2020 and May 2022, 374 subjects were screened for eligibility (**Fig 1**). A total of 294 subjects were excluded, mostly due to participation in another interventional study, or the expectation that postoperative ventilation would last less than two hours. Two subjects withdrew from the study because they were extubated directly upon arrival at the ICU, and thus did not receive postoperative ventilation with the assigned closed–loop ventilation mode. Of the remaining 78 subjects, the surgical and baseline characteristics are shown in **Table 2**. Most of the subjects were male, and the majority underwent either coronary arterial bypass graft surgery or aortic valve surgery. The most common non–cardiac comorbidities were hypertension and diabetes mellitus.

**Table 2:** Baseline characteristics.

	Sidestream capnography (N = 39)	Mainstream capnography (N = 39)
Age, years	65.0 [60.0–70.5]	68.0 [63.0–73.5]
Male gender, %	30/39 (76.9)	26/39 (66.7)
PBW, kg	70.6 [64.7–78.3]	72.4 [63.3–78.3]
BMI, kg/m^2^	26.0 [24.1–29.0]	25.9 [22.6–29.6]
SAPS II score available	39/39 (100.0)	36/39 (92.3)
Median SAPS II score	27.0 [23.5–31.5]	26.0 [23.0–29.0]
EuroSCORE II available	38/39 (97.4)	38/39 (97.4)
Median EuroSCORE II score	1.5 [1.0–2.4]	1.7 [1.0–2.0]
APACHE IV score available	39/39 (100.0)	36/39 (92.3)
Median APACHE IV score	38.0 [33.0–46.0]	40.0 [30.8–44.3]
Smoking status, %		
Never	12/39 (30.8)	14/39 (35.9)
Former (cessation >3 months ago)	19/39 (48.7)	21/39 (53.8)
Current	8/39 (20.5)	4/39 (10.3)
Alcohol use, %		
Never	13/39 (33.3)	11/39 (28.2)
Occasional	16/39 (41.0)	16/39 (41.0)
Frequent	10/39 (25.6)	11/39 (28.2)
Yes, but frequency unclear	0/39 (0.0)	1/39 (2.6)
COPD, %	1/39 (2.6)	1/39 (2.6)
Asthma, %	1/39 (2.6)	1/39 (2.6)
Diabetes Mellitus, %	8/39 (22.2)	7/39 (17.9)
Diet only	1/39 (2.6)	0/39 (0.0)
Oral medication	5/39 (12.8)	3/39 (7.7)
Insulin	2/39 (5.1)	4/39 (10.3)
Hypertension, %	21/39 (53.8)	20/39 (51.3)
OSAS, %	3/39 (7.7)	1/39 (2.6)
Chronic Kidney Injury, %	2/39 (5.2)	0/39 (0.0)
Cerebral Vascular Incident, %	1/39 (2.6)	2/39 (5.1)
Peripheral artery disease, %	3/39 (7.7)	4/39 (10.3)
NYHA classification, %		
Not applicable	10/39 (25.6)	5/39 (12.8)
I	9/39 (23.1)	10/39 (25.6)
II	9/39 (23.1)	13/39 (33.3)
III	9/39 (23.1)	9/39 (23.1)
IV	2/39 (5.1)	2/39 (5.1)
Left Ventricle Ejection Fraction	0.5 [0.5–0.6]	0.5 [0.4–0.5]
Left Ventricle Function, categories		
Reduced (<40%)	3/39 (7.7)	3/39 (7.7)
Mid–range (40–50%)	6/39 (15.4)	10/39 (25.6)
Normal (>50%)	30/39 (76.9)	26/39 (66.7)
Right Ventricle function, categories		
Moderate	2/39 (5.1)	0/39 (0.0)
Good	37/39 (94.9)	39/39 (100.0)
Pulmonary hypertension	1/39 (2.6)	4/39 (10.3)
Atrial Fibrillation	10/39 (25.6)	12/39 (30.8)
Aortic Valve Disease		
None	24/39 (61.5)	18/39 (46.2)
Moderate Insufficiency	9/39 (23.1)	8/39 (20.5)
Severe Insufficiency	4/39 (10.3)	3/39 (7.7)
Moderate Stenosis	1/39 (2.6)	2/39 (5.1)
Severe Stenosis	7/39 (17.9)	14/39 (35.9)
Mitral Valve Disease		
None	23/39 (59.0)	18/39 (46.2)
Moderate Insufficiency	9/39 (23.1)	14/39 (35.9)
Severe Insufficiency	6/39 (15.4)	6/39 (15.4)
Moderate Stenosis	0/39 (0.0)	2/39 (5.1)
Severe Stenosis	1/39 (2.6)	0/39 (0.0)
Tricuspid Valve Disease		
None	31/39 (79.5)	29/39 (74.4)
Moderate Insufficiency	8/39 (20.5)	10/39 (25.6)
Severe Insufficiency	1/39 (2.6)	0/39 (0.0)
Moderate Stenosis	0/39 (0.0)	0/39 (0.0)
Severe Stenosis	0/39 (0.0)	0/39 (0.0)
Perioperative characteristics		
Type of Surgery, %		
CABG	18/39 (46.2)	15/39 (38.5)
Aortic Root	7/39 (17.9)	7/37 (17.9)
Aortic Arch	1/39 (2.6)	3/39 (7.7)
Aortic Valve	13/39 (33.3)	21/39 (53.8)
Mitral Valve	6/39 (15.4)	7/39 (17.9)
Tricuspid Valve	4/39 (10.3)	1/39 (2.6)
Pacemaker	0/39 (0.0)	0/39 (0.0)
Duration of anesthesia, minutes	325.0 [286.3–386.5]	322.0 [278.0–351.0]
Duration of extracorporeal circulation, minutes	122.0 [101.0–162.5]	131.0 [104.0–157.0]
Duration of aortic occlusion, minutes	93.0 [62.5–124.0]	99.0 [61.5–117.0]
First postop CK-MB, ng/mL	26.4 [17.4–42.0]	23.4 [18.6–39.9]

Data are presented as numbers (percentage) or medians [1^st^ quartile–3^rd^ quartile]. For consistency and to enhance readability, we present all our continuous data using the median with interquartile ranges.

Abbreviations: APACHE IV, Acute Physiology and Chronic Health Evaluation Score; BMI, Body Mass Index; CABG, Coronary Artery Bypass Graft; CK-MB, Creatine Kinase MB Isoenzyme; COPD, Chronic Obstructive Pulmonary Disease; OSA, Obstructive Sleep Apnea; PBW, Predicted Bodyweight; NYHA, New York Heart Association; SAPS II, Simplified Acute Physiology Score

### Ventilator parameters

In the first hour after randomization, median RR and FiO_2_ were higher, and median V_T_ was lower in the sidestream capnography group; thereafter, there were no differences between the two groups (**S1**–**S6 Figs**). Compared to sidestream capnography, etCO_2_ measurements during mainstream capnography were higher at all time points (**S7 Fig**).

### Primary outcome

**Fig 2:**
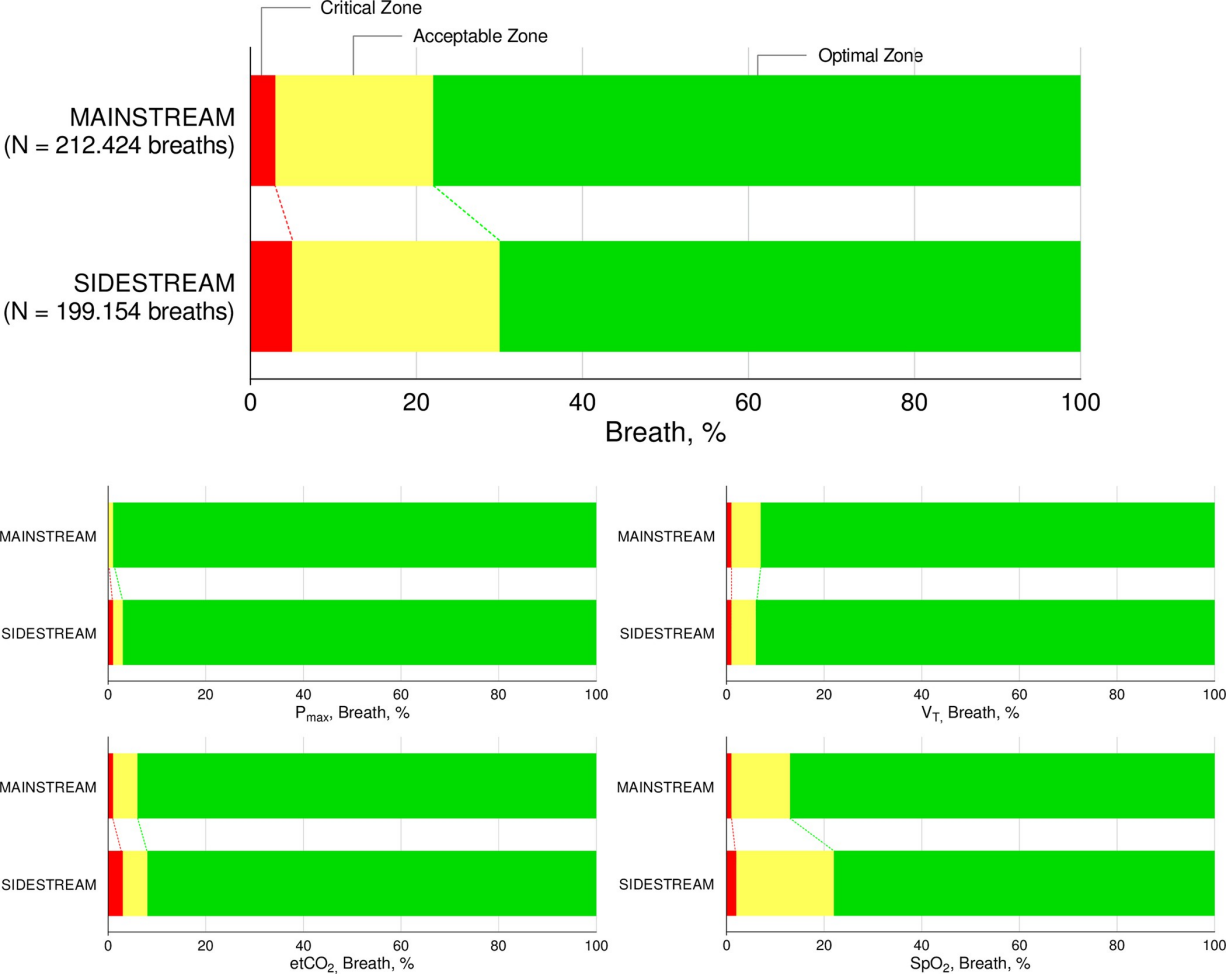
Proportions of breaths in the predefined zones of ventilation.

During the first three hours of postoperative ventilation, the median *proportion of breaths* in the optimal zone of ventilation was 0.77 [IQR, 0.51–0.91] and 0.84 [IQR, 0.67–0.91] in the sidestream capnography and mainstream capnography groups, respectively, (mean ratio 0.87 (one–sided 95% CI 0.77–∞); *P* = 0.116 for non–inferiority), crossing the pre–defined non–inferiority margin of 0.80 (**Fig 2** and **Table 3**). The per protocol analysis did not change this finding (**S1 Table**).

**Table 3:** Primary endpoint and secondary endpoints.

	Sidestream (N = 39)	Mainstream (N = 39)	Mean Ratio (95%–CI)	Absolute Mean Difference (95%–CI)	Odds Ratio (95%–CI)	*P*
**Primary endpoint**						
Proportion of breaths in optimal zone			0.87 (0.77 to ∞)			0.116*
Median [IQR]	0.77 [0.51–0.91]	0.84 [0.67–0.91]				
Mean ± standard deviation	0.69 ± 0.26	0.79 ± 0.16				
**Secondary endpoints**						
Proportion of breaths in acceptable zone						
Median	0.13 [0.08–0.39]	0.15 [0.08–0.27]	1.31 (0.87 to 1.91)			0.181
Mean	0.25 ± 0.23	0.19 ± 0.14				
Proportion of breaths in critical zone						
Median	0.02 [0.01–0.06]	0.01 [0.00–0.03]	2.69 (0.87 to 7.44)			0.072
Mean	0.07 ± 0.14	0.03 ± 0.04				
Proportion of time spent in optimal zone						
Median	0.86 [0.50–0.91]	0.85 [0.71–0.91]				
Mean	0.70 ± 0.24	0.79 ± 0.16		-0.09 (-0.18 to 0.00)		0.057
Proportion of time spent in acceptable zone						
Median	0.13 [0.08–0.38]	0.14 [0.08–0.28]				
Mean	0.24 ± 0.21	0.19 ± .0.14		0.05 (-0.03 to 0.14)		0.203
Proportion of time spent in critical zone						
Median	0.02 [0.01–0.05]	0.01 [0.00–0.03]				
Mean	0.06 ± 0.***12***	0.02 ± 0.04		0.04 (-0.01 to 0.09)		0.101
Proportion of breaths with SpO_2_<85%						
Median	0.00 [0.00–0.00]	0.00 [0.00–0.00]	4.59 (1.23 to 20.42)			0.037
Mean	0.00 ± 0.01	0.00 ± 0.00				
Exact values	247 / 75.327	107 / 82.323				
Duration of postoperative ventilation, hours	4.5 [2.3–5.4]	4.2 [3.0–5.9]		-0.39 (-1.62 to 0.84)		0.532
Duration of weaning, hours	1.2 [0.7–2.5]	1.7 [0.9–3.4]		-0.19 (-0.94 to 0.56)		0.556
Reintubations, %	2 / 39 (5.1)	1 / 39 (2.6)			2.05 (0.19 to 45.31)	0.564
Failed extubations, %	1 / 39 (2.6)	0 / 39 (0.0)			-	1.000
Postoperative Pulmonary Complications, %	5 / 39 (12.8)	7 / 39 (17.9)			0.68 (0.18 to 2.32)	0.532
Atelectasis	2 / 39 (5.1)	0 / 39 (0.0)			-	0.474
Pneumonia	2 / 39 (5.1)	1 / 39 (2.6)			2.05 (0.19 to 45.31)	0.564
Pneumothorax	3 / 36 (7.7)	6 / 39 (15.4)			0.45 (0.09 to 1.88)	0.296
Length of stay in ICU, days	1.0 [1.0–1.0]	1.0 [1.0–1.0]		0.18 (-0.84 to 1.20)		0.728
Length of stay in hospital, days	7.5 [5.6–10.6]	7.6 [6.5–10.5]		1.63 (-1.16 to 4.42)		0.248
Mortality, %						
Mortality at day 30	2 / 39 (5.1)	1 / 39 (2.6)			2.05 (0.19 to 45.31)	0.564
ICU mortality	0 / 39 (0.0)	0 / 39 (0.0)			-	-

Data are presented as numbers (percentage) or medians [1st quartile–3rd quartile]. For consistency and to enhance readability, we present all our continuous data using the median with interquartile ranges.

*P for non-inferiority

Abbreviations: CI, confidence interval; SpO_2_, peripheral oxygen saturation.

### Secondary outcomes

No differences were found between groups for the median *proportion of breaths* spent in the acceptable and critical zones of ventilation. The median *proportion of time* spent in the optimal, acceptable or critical zones of ventilation was also not different between groups. There was a statistically significant difference in the proportion of breaths with hypoxemia between the two groups (0.00 vs. 0.00, mean ratio 4.59 [1.23–20.42], *P* = 0.037). There were no differences in the clinical endpoints (**Table 2** and **S8 Fig**).

### Subgroup analysis

The effect of the used capnography technique on the median proportion of breaths in the optimal zone of ventilation was consistent across the three predefined subgroups (**S9 Fig**).

## Discussion

In this investigator–initiated, single-center, randomized clinical trial we compared the quality of breathing during postoperative ventilation between closed–loop ventilation using sidestream capnography versus mainstream capnography in elective cardiac surgery subjects. We could not confirm the non–inferiority of INTELLiVENT–ASV using sidestream capnography with respect to the proportion of breaths in the predefined optimal zone of ventilation. There was a statistically significant difference in the incidence of breaths with hypoxemia between the groups––this difference disappeared within the first hour of postoperative ventilation. There were no differences in the other endpoints.

This study has several strengths. The trial was performed in a setting where nurses and doctors had extensive experience with the tested closed–loop ventilation mode. The study protocol was straightforward and easy to follow. We used high density, i.e., breath–by–breath ventilator data that was collected directly from the ventilator for the main endpoints. This allowed for an accurate and highly granular comparison of the quality of breathing between the two study groups. The endpoints were predefined and used before [9]. The amount of missing data was low and follow–up was complete. The results of the per–protocol analysis were consistent with the intention–to–treat analysis, adding to the robustness of our findings. Lastly, our study was performed according to recent recommendations pertaining to the reporting of non–inferiority trials [20].

There are several possible explanations for why there were less breaths in the optimal zone in the sidestream group. Sampling delay is a well–known caveat of sidestream capnography, since gas sampling and subsequent transport from the main ventilation circuit to the sensor is required [21]. This is in obvious contrast to mainstream capnography, where the gas mixture is analyzed directly in the circuit. Previous studies in subjects during supine craniotomy and cardiac surgery in the operating room, during noninvasive ventilation in the ICU, and in spontaneously breathing subjects in the emergency room have shown more variable and less accurate etCO_2_ measurements with sidestream capnography [17, 18, 22, 23]. Similar findings come from studies in pediatric populations, especially during periods with higher respiratory rates [24, 25]. Possible reasons for a larger variation in etCO_2_ with sidestream capnography could be due to the addition of inspiratory fresh gas flow to the gas mixture during sampling, or due to variable sampling flow rates during inspiration and expiration due to alternating airway pressures with each breath cycle. These phenomena might also explain the consistently higher etCO_2_ measurements using mainstream capnography in our study, and limit the applicability of sidestream capnography in situations where accurate high–frequency sampling is critical for rapid adjustments in care. However, this may not apply to patients receiving simple postoperative ventilation for a relatively short period of time.

Interestingly, ventilation using sidestream capnography resulted in a higher median RR and a lower median V_T_, early after start of postoperative ventilation. A transiently higher FiO_2_ was also seen in the first hour of ventilation. During closed–loop ventilation the computer automatically adjusts ventilation parameters breath–by–breath based on continuous capnography and pulse oximetry readings. It is conceivable that due to the increased variability in sidestream capnography, the ventilator could have corrected high etCO_2_ measurements by increasing respiratory rate, as the tested closed–loop ventilation mode tries to continue applying lung–protective ventilation using low tidal volumes. The higher RR, however, shortens the inspiratory time, and may even cause a certain level of intrinsic PEEP, and perhaps even some overdistension. The latter could also explain the early differences in oxygenation––it is possible that overdistension led to an increase in perfusion of the dependent lung parts that may have been atelectatic early after surgery, thereby increasing ventilation–perfusion mismatch.

Our study increases the understanding of how the tested closed–loop ventilation mode affects ventilator settings and ventilation parameters during postoperative ventilation. Thus far, only two studies directly compared the quality of breathing between closed–loop ventilation and conventional ventilation in postoperative cardiac surgery subjects [7, 9]. Both studies found closed–loop ventilation to be superior to conventional ventilation with regard to the quality of breathing, using slightly different criteria. In one of these studies, with closed–loop ventilation using mainstream capnography more than two–third of breaths were in the optimal zone of ventilation. We confirmed this finding in our study, in both the mainstream and the sidestream group. Indeed, one silent finding in our study was the large proportion of breaths in the optimal zone, also when using sidestream capnography. The time spent in the critical zone using mainstream capnography was comparable to results of the other study that used comparable definitions for the quality of breathing [7]. Furthermore, in that study conventional ventilation resulted in more time in the critical zone when compared to sidestream capnography in our study. Thus, even though we were not able to verify the non–inferiority of closed–loop ventilation using sidestream when compared to mainstream capnography, closed–loop ventilation using sidestream capnography remains an efficacious and attractive way of providing postoperative ventilation.

This study also has limitations. First, due to the nature of the intervention, caregivers and data collectors could not be blinded for treatment allocation. However, the statistical analysis was performed by an investigator blinded for treatment allocation. Second, this was a single–center trial in a tertiary hospital with extensive experience with closed–loop ventilation. Therefore, it is possible that our findings are not directly generalizable to settings with less experience or less resources. Third, our sample size was not powered to observe differences in secondary clinical endpoints, and all findings should be seen as exploratory. Fourth, as the majority of patients were expected to be extubated within the first 3 hours after start of ventilation in the ICU, we restricted the analysis to the first 3 hours. If the quality of breathing is different at later time points, it could be the subject of a future investigation. Fifth, to increase comparability between patients and homogeneity of the study cohort we adhered to a predefined minimum required time period of postoperative ventilation in the ICU which allowed for an assessment without confounding by duration of ventilation, but this may result in an incomplete reflection of the clinical variability, and with that the quality of breathing with either capnography method. Lastly, as we studied a typical cohort of patients, i.e., one in which ventilation is simple and short–lasting, we cannot give recommendations for use in other patient groups––this applies in particular to patients who benefit from a strict tailoring of the arterial CO_2_ level.

Based on the results of our study, closed–loop ventilation with sidestream capnography should not be preferred over closed–loop ventilation with mainstream capnography in settings where mainstream capnography is widely available, and in subjects wherein mainstream capnography has little or no disadvantages. However, the results of our study should also be interpreted in the context of the non–inferiority design. Moreover, although the non–inferiority of closed–loop ventilation with sidestream could not be established for the quality of breathing, it is uncertain whether this also translates into clinical endpoints.

## Conclusion

This study could not confirm that INTELLiVENT–ASV using sidestream capnography is non–inferior to INTELLiVENT–ASV using mainstream capnography with respect to the quality of breathing in subjects receiving postoperative ventilation after cardiac surgery.

## Supporting information

S1 Checklist CONSORT checklist. (PDF)

S1 File Supplementary methods. (DOCX)

S2 File INTELLiSTREAM trial protocol. (PDF)

S3 File INTELLiSTREAM statistical analysis plan. (DOCX)

S1 Table Primary endpoint and secondary endpoints according to per protocol principle. (DOCX)

S1 Fig Plot of tidal volume (V_T_), Respiratory Rate (RR) and minute ventilation according to capnography group over time. (TIF)

S2 Fig Plot of FiO_2_, etCO_2_ and SpO2 according to capnography group over time. (TIF)

S3 Fig Plot of PEEP, driving pressure (ΔP), plateau pressure (Pplat), maximum airway pressure (Pmax) according to capnography group over time. (TIF)

S4 Fig Cumulative distribution plots of V_T_, RR, MP at 30 minutes after ICU arrival. (TIF)

S5 Fig Cumulative distribution plots of FiO_2_, etCO_2_ and SpO2 at 30 minutes after ICU arrival. (TIF)

S6 Fig Cumulative distribution plots of PEEP, ΔP, Pplat, Pmax at 30 minutes after ICU arrival. (TIF)

S7 Fig Comparison of etCO_2_ measurements between capnography sensors for quality checking. (TIF)

S8 Fig Kaplan Meijer plots for time to weaning, discharge from ICU and hospital. (TIF)

S9 Fig Subgroup analyses on percentage of breaths in predefined optimal zone of ventilation. (TIF)
