# Supplementary material for: Closed–Loop ventilation using sidestream versus mainstream capnography for automated adjustments of minute ventilation—A randomized clinical trial in cardiac surgery patients
Source: PLoS One. 2023 Aug 23;18(8):e0289412. doi: 10.1371/journal.pone.0289412 (PMC10446221; doi:10.1371/journal.pone.0289412)
Supplement: S1 File — (DOCX) [file pone.0289412.s002.docx]

**Closed–loop Ventilation Using Sidestream versus Mainstream Capnography for Automated Adjustments of Minute Ventilation––a randomized clinical trial in cardiac surgery patients**

Sunny G.L.H. Nijbroek*^¶ 1,2^, Jan-Paul Roozeman^¶^ ^1,2^, Sarah Ettayeby^1^, Neeltje M. Rosenberg^1,3^, David M.P. van Meenen^1,2^, Thomas G.V. Cherpanath^1^, Wim K. Lagrand^1^, Robert Tepaske^1^, Robert J.M. Klautz^4,5^, Ary Serpa Neto^1,6,7,8,9^, Marcus J. Schultz^1,10,11^

^¶^These authors contributed equally to this work

^1^Department of Intensive Care, Amsterdam University Medical Centers, location AMC, Amsterdam, the Netherlands

^2^Department of Anesthesiology, Amsterdam University Medical Centers, location AMC, Amsterdam, the Netherlands

^3^Department of Internal Medicine, Spaarne Hospital, Haarlem, the Netherlands

^4^Department of Cardiothoracic Surgery, Amsterdam University Medical Centers, location AMC, Amsterdam, the Netherlands

^5^Department of Cardiothoracic Surgery, Leiden University Medical Center, Leiden, the Netherlands

^6^Australian and New Zealand Intensive Care Research Centre (ANZIC–RC), School of Public Health and Preventive Medicine, Monash University, Melbourne, Australia

^7^Laboratory of Experimental Intensive Care and Anesthesiology (L⋅E⋅I⋅C⋅A), Amsterdam University Medical Centers, location AMC, Amsterdam, the Netherlands

^8^Department of Critical Care, Austin Hospital, Melbourne Medical School, University of Melbourne, Melbourne, Australia

^9^Department of Critical Care Medicine, Hospital Israelita Albert Einstein, Sao Paolo, Brazil

^10^Nuffield Department of Medicine, University of Oxford, Oxford, United Kingdom

^11^Mahidol–Oxford Tropical Medicine Research Unit (MORU), Mahidol University, Bangkok, Thailand

**Correspondence:**

Sunny G.L.H. Nijbroek

Email: [s.g.nijbroek@amsterdamumc.nl](file:///\\\\amc.intra\\data\\group\\divh\\IC-Onderzoek2\\IntelliStream\\Personal%20files\\Manuscript\\submission\\4.%20Plos%20One\\Revisions%20210623\\Manuscript%20draft\\s.g.nijbroek@amsterdamumc.nl) (SN)

# Supplementary Methods

**Period of enrollment**

The first subject was enrolled on July 1^st^ 2020, and the 30 day follow–up for the last subject was completed on May 21^st^ 2022. The authors regretfully acknowledge that this study was not registered to ClinicalTrials.gov before the start of enrolling subjects due to a mistake in communication.

**Data collection and standard treatment of subjects**

Detailed data regarding subject characteristics and comorbidities were recorded after inclusion. After arrival in the ICU, subjects started with closed–loop ventilation using a Hamilton G5 ventilator (Hamilton Medical AG, Bonaduz, Switzerland) as part of standard care after cardiac surgery in our ICU. Depending on treatment allocation mainstream capnography (Capnostat 5 Mainstream CO_2_ Sensor, Respironics Novametrix, LLC, Wallingford, CT) or sidestream capnography (Respironics LoFlo Sidestream CO_2_ Module, Respironics Novametrix, LLC, Wallingford, CT) was used by the ventilator. In all subjects, independent of the type of etCO_2_ monitoring used by the ventilator, mainstream capnography was used for standard monitoring.

All nurses and attending physicians were trained and certified in the use of INTELLiVENT–ASV. Most doctors and nurses had extensive experience with INTELLiVENT–ASV, as this automated ventilation mode is the standard mode of ventilation for patients receiving postoperative ventilation. The attending physicians initiated closed–loop ventilation, typically within 10 minutes after the start of ventilation in the ICU, when the results of the first blood gas analyses became available. The automatic adjustments for percentage of alveolar minute volume, PEEP and FiO_2_ were activated, and standard thresholds were set: PEEP was limited to 5 to 12 cm H_2_O, FiO_2_ was limited to 30 to 100%, and a ‘normal lung condition’ was chosen. In patients with chronic obstructive pulmonary disease (COPD), etCO2 targets could be further adapted to compensate for chronic hypercarbia by choosing the ‘COPD’ lung condition within the INTELLiVENT–ASV mode. In case of a difference between PaCO_2_ and etCO_2_ of ≥ 5 mm Hg, the etCO_2_ target was shifted as described in the clinical guideline in use in the participating ICU. Also, ‘Quick Wean’ and automated spontaneous breathing trials were used to facilitate weaning.

Based on the difference in etCO_2_ and PaCO_2_, the etCO_2_ target could be adapted on the ventilator, and if clinically indicated, the target range for SpO_2_ could also be manually adjusted. Manually switching off one or more of the automated controllers by the attending physician (i.e. due to poor sensor signal quality) was allowed at any point during the study, in compliance with standard ventilation management of the participating ICU. According to the standard operating protocol, in case of a large discrepancy between SpO_2_ and SaO_2_ (SaO_2_ >5% lower than SpO_2_), the automated FiO_2_ and PEEP controllers should be switched off, and in case of a minute ventilation of > 200% of the predicted minute volume, the minute volume controller should be switched off.

Breath–by–breath ventilation data was collected using a so–called Memory Box (Hamilton Medical AG), a data storage device connected directly to the communication port at the ventilator. The quality index for SpO_2_ displayed on the ventilator in use, and was thus also collected by the storage device. Data collection continued until tracheal extubation, or up until a maximum of 6 hours after start of ventilation in the ICU. In subjects that were not weaned from invasive ventilation after 6 hours, ventilation was continued using mainstream capnography which is the standard practice in our ICU.

Subjects were followed up until postoperative day 30. At day 30, subjects were contacted once, to record adverse events, readmissions, and the final date of hospital discharge.

**Subject care not related to the study**

In the operating room subjects were treated according to standard operating procedures for anesthesia and cardiopulmonary bypass (CPB), depending on the type of surgery that was performed. Generally, sevoflurane anesthesia was used and arterial and central venous access, as well as transesophageal echocardiography views were obtained prior to the surgical incision. Anticoagulation for cannulation and CPB was performed using unfractioned heparin, based on activated clotting time measurements. During CPB a minimum mean arterial pressure (MAP) of ≥ 50 mm Hg and Hb of ≥ 4.0 mmol/l was targeted.

In the ICU, subjects were typically cared for by one dedicated board–certified ICU nurse. Changes in treatment were implemented based on observations by the nurse, and according to the recommendations in the local guideline for postoperative care. The local guideline for postoperative care included recommendations on fluid resuscitation with normal saline solutions, blood transfusion to maintain a hemoglobin concentration ≥ 4.3 mmol/L, continuous infusion of norepinephrine to achieve a mean arterial blood pressure ≥ 65 mm Hg, and continuous infusion of dobutamine or milrinone to achieve a cardiac index of ≥ 2.2 L/min/m^2^ or a mixed venous oxygenation of ≥ 60%. Subjects were kept sedated using continuous infusion of propofol until the core temperature reached at least 36.0C, after which sedation was stopped. Subjects received a daily dose of 4 grams of acetaminophen. Additional analgesia was reached with 2 mg boluses of morphine intravenously, as needed. The need for additional analgesia was assessed by the nurse throughout the entire stay. Neuromuscular blocking agents were not used as part of postoperative care in the ICU.


Attending physicians decided to extubate the subject based on general extubation criteria: the subject was responsive and cooperative, had a core temperature > 36.0°C, no uncontrolled arrhythmia, no hemodynamic instability or drain production with a possible need for re–thoracotomy, and adequate ventilation and oxygenation with low ventilation requirements (pressure support < 10 cm H_2_O). T–piece weaning was not used; subjects were extubated once they reached the aforementioned extubation criteria.
