# Supplementary material for: Closed–Loop ventilation using sidestream versus mainstream capnography for automated adjustments of minute ventilation—A randomized clinical trial in cardiac surgery patients
Source: PLoS One. 2023 Aug 23;18(8):e0289412. doi: 10.1371/journal.pone.0289412 (PMC10446221; doi:10.1371/journal.pone.0289412)
Supplement: S2 File — (PDF) [file pone.0289412.s003.pdf]

RESEARCH PROTOCOL

# INTELLiVENT–ASV Using Mainstream versus Sidestream End–Tidal $CO_2$ Monitoring– a Randomized Noninferiority Clinical Trial in Cardiac Surgery Patients

J.P. Roozeman[1], S.G. Nijbroek[2], D.M.P. van Meenen[1,2],
T.G. Cherpanath[1], W.K. Lagrand[1], R. Tepaske[1], R.J.M. Klautz[3,4], A. Serpa Neto[1,5,6],
M.J. Schultz[1,7,8]

**Amsterdam University Medical Centers, location AMC, The Netherlands:**
[1]Department of Intensive Care
[2]Department of Anesthesiology
[3]Department of Cardiothoracic Surgery
[7]Laboratory of Experimental Intensive Care and Anesthesiology (L·E·I·C·A)

**Leiden University Medical Center, The Netherlands:**
[4]Department of Cardiothoracic Surgery

**Hospital Israelita Albert Einstein, São Paulo, Brazil:**
[5]Department of Critical Care Medicine

**Faculdade de Medicina do ABC, São Paulo, Brazil:**
[6]Program of Post–Graduation, Research and Innovation

**Mahidol University, Bangkok, Thailand:**
[8]Mahidol–Oxford Tropical Medicine Research Unit (MORU)

**Correspondence:**
Marcus J. Schultz, MD PhD
Department of Intensive Care, C3–423
Meibergdreef 9
1105 AZ Amsterdam, The Netherlands
E–mail: marcus.j.schultz@gmail.com

## PROTOCOL TITLE

INTELLiVENT–ASV Using Mainstream versus Sidestream End–Tidal CO2 Monitoring– a Randomized Noninferiority Clinical Trial in Cardiac Surgery Patients

| | |
|---|---|
| **Protocol ID** | INTELLiSTREAM |
| **Short title** | Mainstream vs. Sidestream end–tidal $CO_2$–guided INTELLiVENT–ASV |
| **EudraCT number** | N.A. |
| **Version** | 4.0 |
| **Date** | 19 April, 2022 |
| **Coordinating investigators** | Jan-Paul Roozeman<br>Amsterdam University Medical Centers, location AMC<br>Department of Intensive Care, Room C3–423<br>Meibergdreef 9, 1105 AZ Amsterdam, The Netherlands<br>Telephone: +31 20 5669111<br>Email: j.p.roozeman@amsterdamumc.nl<br>&<br>Sunny G.L.H. Nijbroek<br>Amsterdam University Medical Centers, location AMC<br>Department of Anesthesiology, Room G3–228<br>Meibergdreef 9, 1105 AZ Amsterdam, The Netherlands<br>Telephone: +31 20 5666339<br>Email: s.g.nijbroek@amsterdamumc.nl |
| **Principal investigator** | Prof. Marcus J. Schultz<br>Amsterdam University Medical Centers, location AMC<br>Department of Intensive Care, Room C3–423<br>Meibergdreef 9, 1105 AZ Amsterdam, The Netherlands<br>Telephone: +31 20 5662509<br>Email: m.j.schultz@amsterdamumc.nl |
| **Sponsor** | Amsterdam University Medical Centers, location AMC<br>Meibergdreef 9, 1105 AZ Amsterdam, The Netherlands |
| **Subsidizing party** | N.A. |
| **Independent expert** | Dr. J. Horn<br>Amsterdam University Medical Centers, location AMC<br>Meibergdreef 9, 1105 AZ Amsterdam, The Netherlands<br>Telephone: +31 20 5666301<br>Email: j.horn@amsterdamumc.nl |

## PROTOCOL SIGNATURE SHEET

| **Name** | **Signature** | **Date** |
|---|---|---|
| **Head of Department:**<br>Prof. M.B. Vroom | | |
| **Principal Investigator:**<br>Prof. M.J. Schultz | | |

## TABLE OF CONTENTS

LIST OF ABBREVIATIONS AND RELEVANT DEFINITIONS ........ 6
1. INTRODUCTION AND RATIONALE ........ 10
2. OBJECTIVE AND HYPOTHESIS ........ 12
3. STUDY DESIGN ........ 13
4. STUDY POPULATION ........ **Fout! Bladwijzer niet gedefinieerd.**
4.1 Population (base) ........ 14
4.2 Inclusion criteria ........ 14
4.3 Exclusion criteria ........ 14
4.4 Sample size calculation ........ 14
5. TREATMENT OF SUBJECTS ........ 16
5.1 Investigational treatment ........ 16
5.2 Treatment of the patients and standard patient care ........ 17
6. METHODS ........ 19
6.1 Study parameters/endpoints ........ 19
6.1.1 Main study parameter/endpoint ........ 19
6.1.2 Secondary study parameters/endpoints ........ 20
6.2 Randomization, blinding and treatment allocation ........ 21
6.3 Study procedures and data to be collected ........ 21
6.4 Withdrawal of individual subjects ........ 23
6.5 Follow–up of subjects withdrawn from treatment ........ 24
7. SAFETY REPORTING ........ 24
7.1 Temporary halt for reasons of subject safety ........ 24
7.2 AEs, SAEs ........ 24
7.3 Follow–up of adverse events ........ 24
8. STATISTICAL ANALYSIS ........ 25
8.1 Primary study parameter(s) ........ 25
8.2 Secondary study parameter(s) ........ 25
8.3 Other study parameters ........ 26
8.4 Interim analysis ........ 26
9. ETHICAL CONSIDERATIONS ........ 27
9.1 Regulation statement ........ 27
9.2 Recruitment and consent ........ 27
9.3 Objection by minors or incapacitated subjects ........ 27
9.4 Benefits and risks assessment, group relatedness ........ 27
9.5 Compensation for injury ........ 27
10. ADMINISTRATIVE ASPECTS, MONITORING AND PUBLICATION ........ 28
10.1 Handling and storage of data and documents ........ 28
10.2 Monitoring and Quality Assurance ........ 28
10.3 Amendments ........ 28
10.4 Annual progress report ........ 28
10.5 Temporary halt and (prematurely) end of study report ........ 29
10.6 Public disclosure and publication policy ........ 29

11. STRUCTURED RISK ANALYSIS ........ 30
11.1 Potential issues of concern ........ 30
11.2 Synthesis ........ 30
12. REFERENCES ........ 31

## LIST OF ABBREVIATIONS AND RELEVANT DEFINITIONS

| | |
|---|---|
| ΔP | Driving pressure |
| ABR | ABR form, General Assessment and Registration form, is the application form that is required for submission to the accredited Ethics Committee (In Dutch: *Algemene Beoordeling en Registratie*) |
| AE | Adverse Event |
| ARDS | Acute Respiratory Distress Syndrome |
| ASV | Adaptive Support ventilation |
| CCMO | Central Committee on Research Involving Human Subjects (in Dutch: *Centrale Commissie Mensgebonden Onderzoek*) |
| DSMB | Data Safety Monitoring Board |
| $etCO_2$ | End tidal carbon dioxide |
| $FiO_2$ | Fraction of inspired oxygen |
| GCP | Good Clinical Practice |
| $HCO_3^-$ | Bicarbonate |
| ICU | Intensive Care Unit |
| I:E | Ratio between the inspiratory and the expiratory time |
| METC | Medical research ethics committee (MREC); in Dutch: medisch ethische toetsing commissie (METC) |
| MV | Minute volume |
| $PaO_2$ | Arterial oxygen tension |
| $PaCO_2$ | Arterial carbon dioxide tension |
| PCV | Pressure controlled ventilation |
| PEEP | Positive end–expiratory pressure |
| Pplat | Plateau pressure |
| PSV | Pressure support ventilation |
| RASS | Richmond Agitation Sedation Scale |
| RR | Respiratory rate |
| (S)AE | (Serious) Adverse Event |
| $SaO_2$ | Arterial oxygen saturation |
| Sponsor | The sponsor is the party that commissions the organization or performance of the research, for example a pharmaceutical company, academic hospital, scientific organization or investigator. A party that provides funding for a study but does not commission it is not regarded as the sponsor, but referred to as a subsidizing party. |
| WBP | Personal Data Protection Act (in Dutch: *Wet Bescherming Persoonsgevens*) |

| | |
|---|---|
| WMO | Medical Research Involving Human Subjects Act (in Dutch: *Wet Medisch–wetenschappelijk Onderzoek met Mensen*) |

## SUMMARY

**Rationale:** INTELLiVENT–adaptive support ventilation (ASV), a fully automated closed–loop mode of ventilatory support for critically ill patients available on Hamilton intensive care unit (ICU) ventilators, typically uses mainstream end–tidal capnography for automatic adjustments of minute ventilation and other important ventilator settings. Sensors used for mainstream $etCO_2$ measurements are expensive and fragile – sensors for sidestream $etCO_2$ measurements are cheaper and also more robust. Sidestream capnography could serve as an attractive alternative for mainstream capnography with use of INTELLiVENT–ASV.

**Objective**: The objective of this randomized noninferiority trial is to determine whether INTELLiVENT–ASV with sidestream capnography is noninferior to INTELLiVENT–ASV with mainstream capnography with regard to the percentage of breaths in a broadly accepted predefined ‘optimal’ zone of ventilation.

**Study design:** A single–center randomized noninferiority trial.

**Study population:** The study population consists of consecutive elective cardiac surgery patients who are expected to need at least 2 hours of postoperative ventilation in the ICU of the Amsterdam Medical University Centers, location ‘AMC’.

**Intervention:** Shortly after at arrival at the ICU, patients will be randomized to receive either ventilation with INTELLiVENT–ASV with mainstream capnography or with sidestream capnography. Granular ventilation data will be collected until tracheal extubation. Sensors for mainstream and sidestream capnography are readily available and alternately used for $etCO_2$ monitoring in the participating ICU.

**Main study parameters/endpoints:** The co–primary study endpoint is the percentage of breaths a patient spends within the three predefined zones of ventilation during the first three hours of ventilation with INTELLiVENT–ASV.

**Nature and extent of the burden and risks associated with participation, benefit and group relatedness:** Hamilton ventilators can use mainstream and sidestream $etCO_2$ sensors, and both techniques are currently used in the participating ICU. INTELLiVENT–ASV is a safe mode of ventilation, certainly also in patients who receive postoperative ventilation, and therefore this automated mode is preferably used in all cardiac surgery patients in the participating ICU. Of note, as all patients are under sedation during the phase postoperative ventilation is applied, burden for the patient is negligible.

# 1. INTRODUCTION AND RATIONALE

INTELLiVENT–adaptive support ventilation (ASV) is a fully automated closed–loop mode of ventilatory support for intensive care unit (ICU) patients. INTELLiVENT–ASV uses pressure–controlled or pressure support ventilation depending on patient's activity, and continuously adjusts the inspiratory time and pressure levels based on the least work of breathing, which has been shown to result in an adequately low tidal volume ($V_T$) for lung protection.[1–3] In addition, INTELLiVENT–ASV uses pulse oximetry and capnography to adjust positive end–expiratory pressure (PEEP), the fraction of inspired oxygen ($FiO_2$) and minute volume. One recent randomized clinical trial showed INTELLiVENT–ASV to favorably change the time spent in broadly–accepted zones of ventilation in cardiac surgery patients who receive postoperative ventilation in an ICU, when compared to non–automated ventilation [the results of this study are captured in a manuscript presently under review at a peer–reviewed medical journal; ClinicalTrials.gov Identifier: NCT03180203].

INTELLiVENT–ASV can use mainstream as well as sidestream capnography. Mainstream capnography is most often used, as this technique is widely available in modern ICUs. In our hospital, sidestream capnography is mostly used in operating theaters, and at the 'Mobile Intensive Care Unit', where INTELLiVENT–ASV is not yet implemented. Alleged disadvantages of mainstream capnography include increased dead space, possible damage during handling of the sensor, and the additional weight on the airway caused by the sensor block near the endotracheal tube. Alleged disadvantages of sidestream capnography include the risk of accidental crushing or kinking of the sampling tube, and blockage of the tube by condensation from humidified sample gas and airway secretions. As the tools used for sidestream capnography are noticeably cheaper than those used for mainstream capnography,[4,5] sidestream

capnography could serve as an attractive alternative for mainstream capnography with use of INTELLiVENT–ASV. A head–to–head comparison, however, has never been performed.

Whether INTELLiVENT–ASV with sidestream capnography performs as well as INTELLiVENT–ASV with mainstream capnography with respect to quality of breathing is unknown. This study, therefore, will compare INTELLiVENT–ASV with sidestream capnography to INTELLiVENT–ASV with mainstream capnography.

## 2. OBJECTIVE AND HYPOTHESIS

The objective of this randomized noninferiority clinical trial is to compare INTELLiVENT–ASV with sidestream capnography with INTELLiVENT–ASV with mainstream capnography during postoperative ventilation in patients after elective cardiac surgery.

We hypothesize INTELLiVENT–ASV with sidestream capnography is noninferior to INTELLiVENT–ASV with mainstream capnography with respect to the percentage of breaths patient spend within the ‘optimal’ zone of ventilation.

## 3. STUDY DESIGN

'INTELLiSTREAM' is an investigator–initiated, investigator–sponsored, single–center, randomized, noninferiority clinical trial in patients who receive postoperative ventilation in the intensive care unit after elective cardiac surgery.

## 4. STUDY POPULATIONPopulation (base)

The study population consists of consecutive elective cardiac surgery patients who will receive postoperative ventilation in the intensive care unit (ICU).

### 4.2 Inclusion criteria

- Undergoing elective cardiac surgery in the Amsterdam University Medical Centers, location ‘AMC’
- Planned admission to the ICU for postoperative ventilation
- Expected to need postoperative ventilation for at least 2 hours

### 4.3 Exclusion criteria

- Age under 18 years
- Patients previously included in the current clinical trial
- Patients participating in other interventional clinical trials that could influence ventilator settings and ventilation parameters
- Patients with suspected or confirmed pregnancy
- Moribund patients

### 4.4 Sample size calculation

The sample size is computed based on the hypothesis that ventilation with INTELLiVENT–ASV with sidestream capnography is similar to INTELLiVENT–ASV with mainstream capnography with respect to the percentage of breaths within the ‘optimal’ zone of ventilation (see below). Considering an expected percentage of breaths in ‘optimal’ zone with mainstream capnography of 69 ± 23% (results from a previous study, the report of which is currently under review at a peer–reviewed journal; ClinicalTrials.gov Identifier: NCT03180203), a power of 80%, a one–sided alpha level of 0.05 and a non–inferiority margin of 20% (corresponding to 14% less breaths in the

‘optimal’ zone in the sidestream group compared to the mainstream group), 72 patients need to be included.

Patients who are extubated before 90 minutes of postoperative ventilation will be considered drop–outs and will not contribute towards the sample size. This cut–off is chosen to have sufficient time to observe the evolution of postoperative ventilation requirements, as these will significantly change over the first 90 minutes of postoperative ventilation in the ICU. This also increases comparability of patients and reduces the likelihood of clinical and statistical heterogeneity, as patients rapidly awaking from anesthesia after surgery will likely have different respiratory needs. We will continue recruiting patients until both study arms have at least 36 patients with at least 90 minutes of postoperative ventilation per randomization group.

# 5. TREATMENT OF SUBJECTS

## 5.1 Investigational treatment

Upon arrival at the ICU, patients will be randomized to receive either ventilation with INTELLiVENT–ASV with mainstream capnography, or INTELLiVENT–ASV with sidestream capnography (Figure).

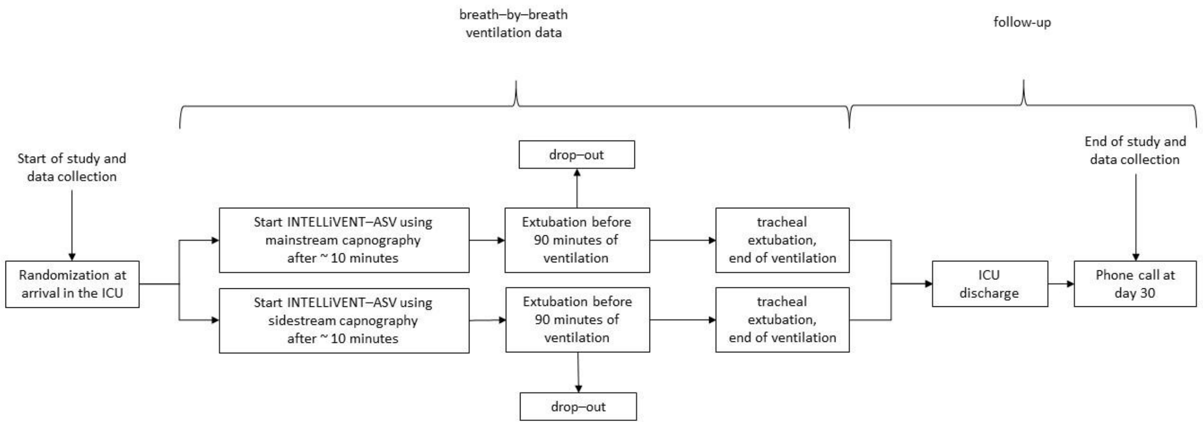


Figure. Study protocol flow chart

The attending physician will initiate INTELLiVENT–ASV, typically within 10 minutes after start of ventilation in the ICU when the results of the first blood gas analyses become available. For this, the attending physician starts the INTELLiVENT–ASV mode, and activates the automatic adjustments for (a) percentage of minute volume, (b) positive end–expiratory pressure (PEEP) and oxygen fraction in inspired air ($FiO_2$) with PEEP limited to between 5 and 12 cm $H_2O$ and $FiO_2$ to between 30 and 100%, and the 'lung condition' set to 'normal'. In case of a difference between $PaCO_2$ and $etCO_2$ of 5 mm Hg or more, the $etCO_2$ target can be shifted as described in the clinical guideline in use in the participating ICU. The two extra options in

INTELLiVENT–ASV, ‘Quick Wean’ and ‘spontaneous breathing trials (SBT), are started when postoperative sedation is stopped (see below).

The tools for mainstream and sidestream capnography necessary for proper functioning of INTELLiVENT–ASV are available in the ICU, and alternately used in daily clinical practice. For the purpose of this trial, the ventilator uses capnography by mainstream or sidestream $etCO_2$ sensors, as per randomization. In all patients, though, mainstream capnography is also used for standard monitoring. Furthermore, although in this trial INTELLiVENT–ASV will be controlled by either sidestream or mainstream capnography, for the purpose of assessing the primary outcome, only data from the mainstream $etCO_2$ sensor will be used in all patients.

## 5.2 Treatment of the patients and standard patient care

Patients are typically cared for by one dedicated ICU nurse, in line with the unit policy. Changes in treatment are implemented based on observations by the ICU nurse, and according to the recommendations in the local guideline for postoperative care.

The local guideline for postoperative care includes recommendations on fluid resuscitation with normal saline and starch solutions, blood transfusion to maintain hemoglobin concentration (≥ 5.0 mmol/L), norepinephrine in continuous infusion to achieve a mean arterial blood pressure ≥ 70 mm Hg, and dobutamine or enoximone to achieve a cardiac index of ≥ 2.5 L/min/$m^2$ or a mixed venous oxygenation of ≥ 60%.

Patients are kept sedated using continuous infusion of propofol until the core temperature reaches at least $36.0^0$C. When core temperature is $36.0^0$C or more, sedation will be stopped. Patients receive a daily dose of 4g of acetaminophen. Additional analgesia is reached with 1 to 2 mg boluses of morphine intravenously, as needed. The need for additional analgesia is assessed by attending ICU nurses throughout the entire ICU stay. Postoperative shivering, if present, is treated with

meperidine (25 mg intravenously). Neuromuscular blocking agents are not used in the postoperative care in the ICU.

Attending physicians will decide to extubate the patient based on general extubation criteria: the patient is responsive and cooperative, has a core temperature > 36.0°C, urine output > 0.5 mL/kg/h, chest tube drainage < 100 mL in the last hour, no uncontrolled arrhythmia or hemodynamic instability, and a respiratory rate of > 10 per minute without machine–controlled breaths for at least 30 minutes. T–piece weaning is not applied; patients are extubated once they reach the aforementioned extubation criteria.

# 6. METHODS

## 6.1 Study parameters/endpoints

### 6.1.1 Main study parameter/endpoint

Three widely accepted and previously used predefined ventilation 'zones' are used, i.e., 'optimal', 'acceptable' and 'critical' (Table 1). The co–primary endpoint is the percentage of breaths a patient spends inside the three predefined ventilation zones during the first 3 hours of postoperative ventilation using INTELLiVENT–ASV. This time frame is chosen as most patients are expected to be extubated soon after the third hour of postoperative ventilation. The three zones are defined as in two previous studies ([6], ClinicalTrials.gov Identifier: NCT03180203) (Table).

Table. Zones of ventilation used to define the primary outcome

| | Critical Zone | Acceptable Zone | Optimal Zone |
|---|---|---|---|
| Tidal volume, ml/kg PBW | > 12 | 8-12 | ≤ 8 |
| | ***OR*** | ***AND/OR*** | ***AND*** |
| Maximum airway pressure, cm $H_2O$ | ≥ 36 | 31 – 36 | ≤ 30 |
| | ***OR*** | ***AND/OR*** | ***AND*** |
| $etCO_2$, mmHg | < 25 OR ≥ 51 | 25 – 30 OR 46 – 51 | 30 – 46 |
| | ***OR*** | ***AND/OR*** | ***AND*** |
| $SpO_2$, % | < 85 | ≥ 98 *OR* 85 – 93 | 93 – 98 *OR* ≥ 93 if $FiO_2$ ≤ 40% |
| **Definitions** | if any parameters present: *'critical zone'* | no parameters in *'critical zone'*, but not all parameters in *'optimal zone'*: *'acceptable zone'* | if all parameters present: *'optimal zone'* |

PBW: predicted body weight; $etCO_2$: end–tidal carbon dioxide by mainstream capnography: $SpO_2$: oxygen saturation by pulse oximetry; $FiO_2$: fraction of inspired oxygen

In case of missing data, if any of the present parameter is within the 'critical' zone, the zone is defined as 'critical'. If data is missing, but none of the parameters are in the 'critical' zone, the zone is defined as 'missing'.

When the capnography signal is lost, INTELLiVENT–ASV 'freezes' minute ventilation, and continues ventilation based on the last registered capnography signal. Ventilation data during signal loss will be analyzed as part of the primary endpoint.

### 6.1.2 Secondary study parameters/endpoints

Secondary endpoints include the percentage of time in the three ventilation zones, during the first 3 hours of postoperative ventilation, time to spontaneous breathing, defined as time from start of ventilation at the ICU until five or more consecutive spontaneous breaths, duration of weaning, defined as time from cessation of sedatives and of a rectal temperature > 35.5ºC to tracheal extubation, duration of postoperative ventilation, defined as time from start of ventilation at the ICU until tracheal extubation, and ventilator parameters ($V_T$, PEEP, maximum airway pressure (Pmax), plateau pressure (Pplat), respiratory rate (RR), minute volume (MV), $FiO_2$, $etCO_2$, $SpO_2$), collected breath–by–breath for 3 hours, as well as the results of clinically–indicated arterial blood gas analysis.

Other outcomes collected are the proportion of failed extubations, defined as re–intubation within 48 hours after extubation and considering only patients who survived and did not undergo re–sternotomy during this time, development of postoperative pulmonary complications, a collapsed composite of pneumonia, pneumothorax or severe atelectasis, ICU length of stay and ICU readmission, ICU– and 30–day mortality, capnography equipment signal loss requiring a correction by ICU nurses, and incidence of hypoxemia, defined as percentage of breaths with $SpO_2$ < 85% but only when $SpO_2$ had a quality index > 50%.

## 6.2 Randomization, blinding and treatment allocation

Included patients will be randomly allocated in a 1:1 ratio to start ventilation with INTELLiVENT–ASV using either a mainstream or a sidestream capnography. The allocation sequence will be computer–generated using permuted blocks of random block sizes. Randomization will then be performed by local investigators patient-by patient using a dedicated, password protected, SSL–encrypted website. Patients will remain unaware of which sensor technique for capnography was used to control the ventilator. Due to the nature of the treatment, blinding of the care providers is not possible. However, the outcome assessor analyzing the results will remain blinded to treatment allocation.

## 6.3 Study procedures and data to be collected

Patients will receive postoperative ventilation using INTELLiVENT–ASV as part of standard care after cardiac surgery. Data will be collected while the patient is receiving postoperative ventilation in the intensive care unit (ICU). Collection of data will continue until tracheal extubation, or until 6 hours after start of ventilation in the ICU, whatever comes first. Patients randomized to sidestream capnography will be placed on mainstream capnography after 6 hours of ventilation in the ICU, if not yet extubated.

In case the capnography signal is lost, e.g. due to kinking or obstruction of the tube with sidestream capnography, or sensor disconnection or condense in the sensor-eye, INTELLiVENT–ASV ‘freezes’ the minute ventilation, and continues ventilation based on the last registered capnography signal, guaranteeing minute volume and patient safety. At the same moment, a visual and auditory alarm is activated, after which the nurse will solve the problem, and INTELLiVENT–ASV restarts using the capnography signal to adjust the minute volume.

*Before surgery*

- Gender and age (male + years)
- Height and weight (kg + cm)
- Type of cardiac surgery
- PBW, kg
- BMI, kg/m2
- SAPS II
- APACHE IV
- EuroSCORE II
- Smoking (no, current, former)
- Use of alcohol
- COPD
- Asthma
- Obstructive sleep apnea (OSA)
- Diabetes mellitus
- Hypertension
- CVA or TIA
- NYHA classification
- Peripheral artery disease
- Chronic kidney disease
- Left ventricular ejection fraction (LVEF)
- Right ventricular function
- Aortic valve disease
- Mitral valve disease
- Tricuspid valve disease

- Duration of extracorporeal circulation, minutes
- Duration of aortic occlusion, minutes
- First postoperative level of CK–MB, U/L

*After surgery, at arrival in the ICU and till end of ventilation*

- Tidal volume ($V_T$, ml/kg PBW) (breath–by–breath)
- Positive end–expiratory pressure (PEEP, cm $H_2O$) (breath–by–breath)
- Maximum airway pressure (Pmax, cm $H_2O$) (breath–by–breath)
- Respiratory rate (RR, per minute) (breath–by–breath)
- Fraction of inspired oxygen ($FiO_2$, %) (breath–by–breath)
- End–tidal $CO_2$ (et–$CO_2$, mm Hg) (breath–by–breath)
- Pulse oximetry (SpO2, %) (breath–by–breath)

*And, if available, from clinically–indicated blood gas analyses*

- Partial arterial $CO_2$ pressure ($PaCO_2$, mm Hg)
- Partial arterial oxygen pressure ($PaO_2$, mm Hg)
- Arterial oxygen saturation ($SaO_2$, %)
- Arterial pH
- Arterial bicarbonate level ($HCO_3^-$, mmol/L)
- Arterial lactate level

## 6.4 Withdrawal of individual subjects

The investigator can decide to withdraw a subject from the study for urgent medical reasons. Subjects can decide to retract informed consent before start of anesthesia for surgery, and also to retract consent for use of data after tracheal extubation. In the randomization log these cases will be recorded without patient–specific data.

## 6.5 Follow–up of subjects withdrawn from treatment

Patients will be subjected to a 30-day follow-up after postoperative admission to the ICU. Patients will be contacted once, 30 days after inclusion, to assess for complications, readmissions, and the final date of hospital discharge.

# 7. SAFETY REPORTING

## 7.1 Temporary halt for reasons of subject safety

In accordance to section 10, subsection 4, of the WMO, the sponsor will suspend the study if there is sufficient ground that continuation of the study will jeopardize subject health or safety. The sponsor will notify the accredited METC without undue delay of a temporary halt including the reason for such an action. The study will be suspended pending a further positive decision by the accredited METC. The investigator will take care that all subjects are kept informed.

## 7.2 AEs, SAEs

This study compares fully automated ventilation with two types of sensors that are used within their indication, and no related serious adverse events (SAEs) are expected. Related SAEs or possible related SAEs will be reported to the METC within 7 days of first knowledge of SAE. Unrelated SAEs will be reported to the METC via line listing once per year. All SAEs and related AEs will be recorded in the eCRF.

## 7.3 Follow–up of adverse events

Follow-up of AEs and SAEs is 30 days after the intervention.

# 8. STATISTICAL ANALYSIS

## 8.1 Primary study parameter(s)

The primary outcome, the percentage of breaths spent in the predefined 'optimal' zone, will be analyzed for non–inferiority, considering the margin of non–inferiority of 20%. Data will be collected breath-by-breath, but the percentage of breaths in the 'optimal' zone will be summarized per patient according to the formula below (Eq.1).

Data will be presented as median (quartile 25% - quartile 75%) and mean ± standard deviation, compared as a mean ratio (as described in the Eq. 2), tested for non–inferiority considering a margin of 20%, and presented as a one–sided 95% confidence interval. Thus, non–inferiority will be established if lower boundary of the one–sided 95% confidence interval was higher than 0.80 (20% decrease in percentage of breaths in optimal zone). A one–sided *p* value for non–inferiority will be calculated. Results will be presented in a table of outcomes and also in a forest plot. Statistical uncertainty will be expressed by 95% confidence intervals.

$$\%breaths_{optimal} = \left(\frac{optimal_{numberbreaths}}{total_{numberbreaths}}\right) * 100\ (Eq.1)$$

$$Mean\ Ratio = \frac{Mean_{\%breathsoptimal}\ in\ Sidestream}{Mean_{\%breathsoptimal}\ in\ Mainstream} > 0.80\ (Eq.2)$$

All statistical analyses will be performed with the R language and environment for statistical computing.

## 8.2 Secondary study parameter(s)

For the analyses of ventilatory parameters over the first three hours of ventilation, all parameters will be summarized as the mean of every 5 minutes until extubation or 180 minutes, whichever comes first. The groups will be compared using mixed–effect longitudinal models with patients as random effect, the variable of interest as the dependent variable and the time of measurement, randomization group and an

interaction of time and randomization group as fixed effects. Two *p* values will be reported: 1) *p* value for the group difference, reflecting the overall test for difference between groups across the three hours; and 2) *p* values for the group x time interaction, evaluating if change over time differed by group. In addition, since it is expected that the baseline values will be similar between the groups, these will be exposed in the graphs but excluded from the models.

For outcomes assessing proportions of breaths and incidence of hypoxemia, the denominator will be the total number of breaths. Secondary binary outcomes, including the proportions of breaths and incidence of hypoxemia, will be assessed with risk ratio and 95%–CIs calculated with Wald likelihood ratio approximation test and $\chi 2$ tests for hypothesis testing. The effects of the intervention on time to spontaneous breathing, duration of weaning and ventilation, time until ICU discharge and 30–day mortality will be assessed using Kaplan–Meier survival curves and reported as hazard ratios with 95%–CIs calculated from a Cox proportional hazard model. The Schoenfeld residuals against the transformed time will be used to test the proportional hazard assumptions. Survival time will be calculated from time of randomization until time of the outcome. Analyses will be performed with R statistics version 3.0.2. Patient characteristics will be compared and described using appropriate statistics.

## 8.3 Other study parameters

N.A.

## 8.4 Interim analysis

N.A.

# 9. ETHICAL CONSIDERATIONS

## 9.1 Regulation statement

This study will be conducted according to the principles of the Declaration of Helsinki as stated in the current version of Fortaleza, Brazil, 2013 and in accordance with the Medical Research Involving Human Subjects Act (WMO).

## 9.2 Recruitment and consent

Patients will be recruited at pre-assessment by the anesthesiologist. Subjects will be informed verbally by local researchers, and by a patient information letter. The patient will be given sufficient time to consider their decision and to discuss the decision with their relatives or the independent expert. Written informed consent will be obtained prior to surgery, with the possibility to ask any remaining questions about participation.

## 9.3 Objection by minors or incapacitated subjects

N.A.

## 9.4 Benefits and risks assessment, group relatedness

Both mainstream and sidestream $etCO_2$ sensors are used within their indication. This entails no increased risk. Furthermore, during the study all patients will be sedated as part of standard care, this minimalizes the burden for the patient.

## 9.5 Compensation for injury

The sponsor/investigator has an insurance for liability, which is in accordance with article 7 subsection 6 of the WMO. All research subjects are insured to cover damage through injury or death caused by the study. The insurance applies to the damage that becomes apparent during the study or within 4 years after end of the study.

# 10. ADMINISTRATIVE ASPECTS, MONITORING AND PUBLICATION

## 10.1 Handling and storage of data and documents

All patients will be assigned a patient identification code. The codebook will be stored digitally and in paper. The paper version will be stored in a locked cabinet and the digital form will be encrypted with a double password. All data will be stored for the length of the study and for 15 years afterwards. All handling of personal data will comply with the GDPR.

## 10.2 Monitoring and Quality Assurance

Queries on the database will be done by a statistician and analyzed by the monitor to signalize early aberrant patterns, trends and issues with consistency or credibility. On–site monitoring will comprise controlling presence and completeness of the research dossier and the informed consent forms. Independent on-site monitoring will be performed by the Clinical Research Unit of the Amsterdam UMC location AMC.

## 10.3 Amendments

Amendments are changes made to the research protocol after a favorable opinion by the accredited METC has been given. All amendments will be notified to the METC that gave a favorable opinion. All substantial amendments will be notified to the METC and to the competent authority. Non–substantial amendments will not be notified to the accredited METC and the competent authority, but will be recorded and filed by the sponsor.

## 10.4 Annual progress report

The sponsor/investigator will submit a summary of the progress of the trial to the accredited METC once a year. Information will be provided on the date of inclusion of the first subject, numbers of subjects included and numbers of subjects that have

completed the trial, serious adverse events/ serious adverse reactions, other problems, and amendments.

### 10.5 Temporary halt and (prematurely) end of study report

The investigator will notify the accredited METC of the end of the study within a period of 8 weeks. In case the study is ended prematurely, the investigator will notify the accredited METC within 15 days, including the reasons for the premature termination. Within one year after the end of the study, the investigator/sponsor will submit a final study report with the results of the study, including any publications/abstracts of the study, to the accredited METC.

### 10.6 Public disclosure and publication policy

The results of the study will find their way into (inter–) national scientific journals and guidelines. We will submit the analyses to scientific journals in the field of intensive care medicine as well as anesthesiology, since both ICU physicians and anesthesiologists apply ventilation in the ICU setting.

# 11. STRUCTURED RISK ANALYSIS

## 11.1 Potential issues of concern

Chapter 12.1 is skipped because both types of $etCO_2$ sensors are used within their indication, and are alternately used in the participating unit.

## 11.2 Synthesis

There is no additional risk to mechanical ventilation with INTELLiVENT–ASV with sidestream or mainstream capnography, other than the risks associated with ventilation per se. Patients included in this study will receive mechanical ventilation as part of standard care. Therefore, this intervention does not lead to an increase of any risk for the patient. Extubation will not be delayed because of this study. Furthermore, patients will be sedated during the study, without exception.

## 12. REFERENCES


1. Garnero, A. *et al.* Routine Use Of A Fully Close Loop Ventilation Mode In Long Term Ventilated ICU Patients: A Prospective Study. in A3188–A3188 (American Thoracic Society, 2012). doi:10.1164/ajrccm-conference.2012.185.1_MeetingAbstracts.A3188
2. Sulemanji, D. S., Marchese, A., Wysocki, M. & Kacmarek, R. M. Adaptive support ventilation with and without end-tidal CO2 closed loop control versus conventional ventilation. *Intensive Care Med.* **39,** 703–10 (2013).
3. Clavieras, N. *et al.* Prospective Randomized Crossover Study of a New Closed-loop Control System versus Pressure Support during Weaning from Mechanical Ventilation. *Anesthesiology* **119,** 631–641 (2013).
4. Chan, K. L., Chan, M. T. V & Gin, T. Mainstream vs. sidestream capnometry for prediction of arterial carbon dioxide tension during supine craniotomy. *Anaesthesia* **58,** 149–55 (2003).
5. Pekdemir, M., Cinar, O., Yilmaz, S., Yaka, E. & Yuksel, M. Disparity between mainstream and sidestream end-tidal carbon dioxide values and arterial carbon dioxide levels. *Respir. Care* **58,** 1152–6 (2013).
6. Lellouche, F., Bouchard, P.-A., Simard, S., L’Her, E. & Wysocki, M. Evaluation of fully automated ventilation: a randomized controlled study in post-cardiac surgery patients. *Intensive Care Med.* **39,** 463–471 (2013).
