# Supplementary material for: Closed–Loop ventilation using sidestream versus mainstream capnography for automated adjustments of minute ventilation—A randomized clinical trial in cardiac surgery patients
Source: PLoS One. 2023 Aug 23;18(8):e0289412. doi: 10.1371/journal.pone.0289412 (PMC10446221; doi:10.1371/journal.pone.0289412)
Supplement: S3 File — (DOCX) [file pone.0289412.s004.docx]

**Statistical Analysis Plan of**

**the noninferiority INTELLiSTREAM trial**

**Rationale**

INTELLiVENT–adaptive support ventilation (ASV) is a fully automated closed–loop mode of ventilatory support for intensive care unit (ICU) patients. INTELLiVENT–ASV uses pressure–controlled or pressure support ventilation depending on patient’s activity, and continuously adjusts the inspiratory time and pressure levels based on the least work of breathing, which has been shown to result in an adequately low tidal volume (VT) for lung protection. In addition, INTELLiVENT–ASV uses pulse oximetry and capnography to adjust positive end–expiratory pressure (PEEP), the fraction of inspired oxygen (FiO2) and minute volume. One recent randomized clinical trial showed INTELLiVENT–ASV to favorably change the time spent in broadly–accepted zones of ventilation in cardiac surgery patients who receive postoperative ventilation in an ICU, when compared to non–automated ventilation [1]. INTELLiVENT–ASV can use mainstream as well as sidestream capnography. Mainstream capnography is most often used, as this technique is widely available in modern ICUs. In our hospital, sidestream capnography is mostly used in operating theaters, and at the ‘Mobile Intensive Care Unit’, where INTELLiVENT–ASV is not yet implemented. Alleged disadvantages of mainstream capnography include increased dead space, possible damage during handling of the sensor, and the additional weight on the airway caused by the sensor block near the endotracheal tube. Alleged disadvantages of sidestream capnography include the risk of accidental crushing or kinking of the sampling tube, and blockage of the tube by condensation from humidified sample gas and airway secretions. As the tools used for sidestream capnography are noticeably cheaper than those used for mainstream capnography, sidestream could be an attractive alternative.

**Aim of this investigation**

To compare INTELLiVENT-ASV with sidestream to INTELLiVENT-ASV with mainstream during postoperative ventilation in patients after cardiac surgery.

**Hypothesis**

INTELLiVENT-ASV with sidestream capnography is noninferior to INTELLiVENT-ASV with mainstream capnography with respect to quality of breathing, i.e. the percentage of breaths patient spend within the ‘optimal zone’ of ventilation.

**Sample size calculation**

The sample size is computed considering an expected percentage of breaths in ‘optimal’ zone with mainstream capnography of 69 ± 23% [1] , a power of 80%, a one –sided alpha level of 0.05 and a non–inferiority margin of 20% (corresponding to 14% less breaths in the ‘optimal’ zone in the sidestream group compared to the mainstream group), 72 patients need to be included.

Patients who are extubated before 90 minutes of postoperative ventilation will be considered drop–outs and will not contribute towards the sample size. This cut–off is chosen to have sufficient time to observe the evolution of postoperative ventilation requirements, as these will significantly change over the first 90 minutes of postoperative ventilation in the ICU. This also increases comparability of patients and reduces the likelihood of clinical and statistical heterogeneity, as patients rapidly awaking from anesthesia after surgery will likely have different respiratory needs. We will continue recruiting patients until both study arms have at least 36 patients with at least 90 minutes of postoperative ventilation per randomization group.

**Patients**

Patients are randomized in a 1:1 ratio to either INTELLiVENT-ASV with sidestream, the ‘Sidestream group’, or to INTELLiVENT-ASV with mainstream, the ‘Mainstream. group.’ The CONSORT flowchart is shown in **Dummy Figure 1** (below)


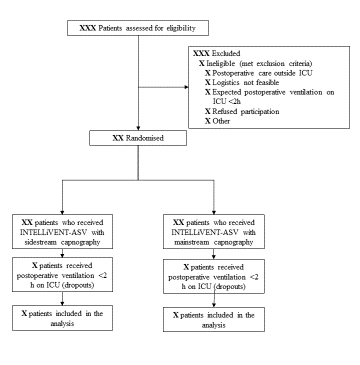


**Dummy Figure 1 - Consort Flowchart**

**Study endpoints**

The primary endpoint is the proportion of breaths spent inside the predefined ‘optimal’ zone during the first 3 hours of postoperative ventilation using INTELLiVENT-ASV (**Dummy Table 1**). Secondary endpoints include:

- The proportion of breaths within each predefined ventilatory zone in the first 3 hours of postoperative ventilation; outcomes regarding proportion of time spent in the three zones of ventilation were also using the complete ventilation time instead of the first three postoperative hours.
- Time to spontaneous breathing, defined as time from start of ventilation at the ICU until five or more consecutive spontaneous breaths
- Duration of weaning, defined as time from cessation of sedative and of rectal temperature > 35.5C to tracheal extubation
- Duration of postoperative ventilation, defined as time from start of ventilation at ICU until tracheal extubation
- Proportion of failed extubations, defined as re-intubation within 48 hours after extubation, excluding patients for re-sternotomy
- Development of postoperative pulmonary complications, a collapsed composite of
  - pneumonia, defined as a patient receiving antibiotics and meets at least one of the following criteria: new or changed sputum, new or changed lung opacities on chest radiography when clinically indicated, tympanic temperature > 38.3C, white blood cell count 12,000/mm^3;
  - pneumothorax, defined as air in the pleural space with no vascular bed surrounding the visceral pleura on chest radiography; or
  - severe atelectasis, defined as lung opacification with a shift of the mediastinum, hilum or hemi diaphragm towards the affected area, and compensatory over-inflation in the adjacent non-atelectatic lung on chest radiography.
- ICU length of stay and readmission
- ICU and 30-day mortality

| **Dummy Table 1 - Zones of ventilation used to define the primary outcome as defined in previous studies [de Bie et al; Lelouch et al]** | | | | | | |
| --- | --- | --- | --- | --- | --- | --- |
|  | **Optimal Zone** | | **Acceptable Zone** | **Critical Zone** | | |
| Tidal volume, ml/kg PBW | | ≤ 8 | 8-12 | | > 12 | |
|  | | ***AND*** | ***AND/OR*** | | | ***OR*** |
| Maximum airway pressure, cm H2O | | ≤ 30 | 31 – 36 | | ≥ 36 | |
|  | | ***AND*** | ***AND/OR*** | | | ***OR*** |
| etCO2, mmHg | | 30 – 46 | 25 – 30 OR 46 – 51 | | < 25 OR ≥ 51 | |
|  | | ***AND*** | ***AND/OR*** | | | ***OR*** |
| SpO2, % | | 93 – 98 *OR* ≥ 93 if FiO2 ≤ 40% | ≥ 98 *OR* 85 – 93 | | < 85 | |
| Definitions | | if all parameters present:  *‘optimal zone’* | no parameters in *‘critical zone’*, but not all parameters in *‘optimal zone’:*  *‘acceptable zone’* | | if any parameters present:  *‘critical zone’* | |
| PBW: predicted body weight; etCO2: end–tidal carbon dioxide by mainstream capnography: SpO2: oxygen saturation by pulse oximetry; FiO2: fraction of inspired oxygen | | | | | | |

**Baseline descriptive characteristics**

Patients will be stratified according to randomization group in sidestream versus mainstream. Patient baseline, surgery and ventilation characteristics will be presented as shown in **dummy Table 2.** The predicted bodyweight of male patients will be calculated as 50+0.91(height [cm] -152.4) and for female patients as 45.5+0.91(height [cm]-152.4).

| **Dummy Table 2 – Baseline characteristics of the included patients** | | |
| --- | --- | --- |
|  | **Sidestream**  **(*n* = XX)** | **Mainstream**  **(*n* = XX)** |
| Age, years |  |  |
| Male gender |  |  |
| PBW, kg |  |  |
| BMI, kg/m^2^ |  |  |
| SAPS II |  |  |
| APACHE IV |  |  |
| EuroSCORE II |  |  |
| Smoking |  |  |
| No |  |  |
| Current |  |  |
| Former |  |  |
| Use of alcohol |  |  |
| COPD |  |  |
| Asthma |  |  |
| OSA |  |  |
| Diabetes mellitus |  |  |
| Hypertension |  |  |
| CVD or TIA |  |  |
| Heart failure |  |  |
| NYHA classification |  |  |
| I |  |  |
| II |  |  |
| III |  |  |
| IV |  |  |
| Peripheral artery disease |  |  |
| Chronic kidney disease, % |  |  |
| LVEF |  |  |
| Right ventricular function |  |  |
| Good |  |  |
| Moderate |  |  |
| Poor |  |  |
| Aortic valve disease |  |  |
| None |  |  |
| Moderate insufficiency |  |  |
| Severe insufficiency |  |  |
| Moderate stenosis |  |  |
| Severe stenosis |  |  |
| Mitral valve disease |  |  |
| None |  |  |
| Moderate insufficiency |  |  |
| Severe insufficiency |  |  |
| Severe stenosis |  |  |
| Tricuspid valve disease |  |  |
| None |  |  |
| Moderate insufficiency |  |  |
| Severe insufficiency |  |  |
| Type of surgery |  |  |
| CABG |  |  |
| Valve surgery |  |  |
| CABG + Valve surgery |  |  |
| Off-pump CABG |  |  |
| Aortic repair |  |  |
| Myxoma excision |  |  |
| Duration of extracorporeal circulation, minutes |  |  |
| Duration of aortic occlusion, minutes |  |  |
| First postoperative level of CK–MB, U/L |  |  |
| Data are median (25 – 75% quartile) or No (%)  *PBW: predicted body weight; BMI: body mass index; SAPS: Simplified Acute Physiology Score; APACHE: Acute Physiology and Chronic Health Evaluation; COPD: chronic obstructive pulmonary disease; OSA: obstructive sleep apnea; CVD: cerebrovascular disease; TIA: transient ischemic attack; NYHA: New York Heart Association; LVEF: left-ventricular ejection fraction; CABG: coronary artery bypass graft; CK-MB: Creatine Kinase MB Isoenzyme* | | |

**Analysis of the primary outcome**

Primary and secondary endpoints will be presented in **dummy Table 3** (see below)**.** The primary outcome, the percentage of breaths spent in the predefined ‘optimal’ zone, will be analyzed for non–inferiority, considering the margin of non–inferiority of 20%. Data will be collected breath-by-breath, but the percentage of breaths in the ‘optimal’ zone will be summarized per patient according to the formula below (Eq.1).

Data will be presented as median (quartile 25% - quartile 75%) and mean ± standard deviation, compared as a mean ratio (as described in the Eq. 2), tested for non–inferiority considering a margin of 20%, and presented as a one–sided 95% confidence interval. Thus, non–inferiority will be established if lower boundary of the one–sided 95% confidence interval was higher than 0.80 (20% decrease in percentage of breaths in optimal zone). A one–sided *p* value for non–inferiority will be calculated. Results will be presented in a table of outcomes and also in a forest plot. Statistical uncertainty will be expressed by 95% confidence intervals.

$$\%breaths_{optimal}= \left( \frac{{optimal}_{numberbreaths}}{total_{numberbreaths}} \right)*100 (Eq.1)$$

$$Mean Ratio= \frac{{Mean}_{\%breathsoptimal} in Sidestream}{{Mean}_{\%breathsoptimal} in Mainstream}>0.80 (Eq. 2)$$

**Analysis of the secondary outcomes**

For the analyses of ventilatory parameters over the first three hours of ventilation, all parameters will be summarized as the mean of every 5 minutes until extubation or 180 minutes, whichever comes first. The groups will be compared using mixed–effect longitudinal models with patients as random effect, the variable of interest as the dependent variable and the time of measurement, randomization group and an interaction of time and randomization group as fixed effects. Two *p* values will be reported: 1) *p* value for the group difference, reflecting the overall test for difference between groups across the three hours; and 2) *p* values for the group x time interaction, evaluating if change over time differed by group. In addition, since it is expected that the baseline values will be similar between the groups, these will be exposed in the graphs but excluded from the models.

For outcomes assessing proportions of breaths and incidence of hypoxemia, the denominator will be the total number of breaths. Secondary binary outcomes, including the proportions of breaths and incidence of hypoxemia, will be assessed with risk ratio and 95%–CIs calculated with Wald likelihood ratio approximation test and χ2 tests for hypothesis testing. The effects of the intervention on time to spontaneous breathing, duration of weaning and ventilation, time until ICU discharge and 30–day mortality will be assessed using Kaplan–Meier survival curves and reported as hazard ratios with 95%–CIs calculated from a Cox proportional hazard model. The Schoenfeld residuals against the transformed time will be used to test the proportional hazard assumptions. Survival time will be calculated from time of randomization until time of the outcome. Analyses will be performed with R statistics version 3.0.2. Patient characteristics will be compared and described using appropriate statistics.

| **Dummy Table 3 – Co-primary and secondary outcomes** | | | | |
| --- | --- | --- | --- | --- |
|  | **Sidestream**  **(*n* = xx)** | **Mainstream**  **(*n* = xx)** | **Effect Estimate**  **(95% CI)** | ***p value^≠^*** |
| **Co-primary outcomes** |  |  |  |  |
| Percentage of breaths in the critical zone^*^  Median (IQR) |  |  |  |  |
| Percentage of breaths in the acceptable zone^*^  Median (IQR) |  |  |  |  |
| Percentage of breaths in the optimal zone^*^  Median (IQR) |  |  |  |  |
| **Secondary outcomes** |  |  |  |  |
| Percentage of time in the critical zone^**^ |  |  |  |  |
| Percentage of time in the acceptable zone^**^ |  |  |  |  |
| Percentage of time in the optimal zone^**^ |  |  |  |  |
| Time until spontaneous breathing, minutes^££^  Median (IQR) |  |  |  |  |
| Duration of weaning, minutes^£^  Median (IQR) |  |  |  |  |
| Duration of ventilation, minutes^****^  Median (IQR) |  |  |  |  |
| Proportion of failed extubations^***^ |  |  |  |  |
| Incidence of postoperative pulmonary complications  Pneumonia  Pneumothorax  Atelectasis |  |  |  |  |
| ICU length of stay, days  Median (IQR) |  |  |  |  |
| ICU readmissions^£££^ |  |  |  |  |
| Mortality  ICU  30-day |  |  |  |  |
| Percentage of breaths with SpO_2_ < 85%^¥,**^ |  |  |  |  |
| ^*^ during the first three hours of ventilation or until extubation and reported according to the total number of breaths  ^**^ during the first three hours of ventilation or until extubation and for at least 30 consecutive seconds  ^***^ defined as any re-intubation within 48 hours after extubation and considering only patients who survived and did not undergo a re-sternotomy during this time  ^****^ time from ICU admission until first successful extubation  ^£^ time from stopping sedatives until successful extubation  ^££^ time from ICU admission until ≥ 5 consecutives spontaneous breaths  ^£££^ during the first 72 hours after ICU discharge  ^¥^ when the measured SpO_2_ had a quality index > 50%  ***^≠^*** *P* value for noninferiority  ^a^ effect estimate is mean difference  ^b^ effect estimate is risk ratio  ^c^ effect estimate is hazard ratio  ^d^ since no event was observed in one group, the effect estimate was not calculated (infinite in the upper limit)  ^e^ calculated with Fisher exact test  ^f^ calculated with log-rank test | | | | |

**Per protocol analysis**

In creating a per protocol analysis, we will exclude patients who had one or more major protocol violations (not meeting minimum ventilation time requirement). Patients receiving the mode of the group that they were not assigned to will be excluded for the per protocol analysis of the primary outcome. The patient will be included for the per protocol analysis of the primary endpoint during the first three hours, if the change in mode occurred after the first three hours of ventilation. Patients that had died, or had a re-sternotomy (for example due to bleeding) during the first three hours (for the first three hours per protocol analysis) or during the logging of the Memorybox (for the overall recorded per protocol analysis) will be excluded for the per protocol analysis of the primary outcome.

**Intention to treat**

In creating intention to treat analysis, we will analyze the recorded ventilation data according to the treatment they actually received. Patients receiving the mode of the group that they were not assigned to will be excluded for the per protocol analysis of the primary endpoint.

**Subgroup analyses**

Treatment effect on primary outcome will be analyzed according to the following subgroups: 1) patients wo were successfully extubated before or after the median postoperative mechanical ventilation time; 2) based on the intraoperative mechanical ventilation time (shorter or longer than the median); and 3) patients with PA2O / FiO2 below or above the median at admission in the ICU. The effect on subgroups will be evaluated according to the interaction effects between each subgroup and the study arms by generalized linear models considering zero-inflated distributions and presented in a forest plot.

**Conclusion**

According to best research practice, we reported here the pre–specified detailed statistical analysis plan prior to locking the database and starting analyses. This document guarantees against reporting bias, selective reporting and data–driven results, as such enhancing the utility of the reported results.

**References**

1. De Bie AJR, Neto AS, van Meenen DM, Bouwman AR, Roos AN, Lameijer JR, et al. Fully automated postoperative ventilation in cardiac surgery patients: a randomised clinical trial. Br J Anaesth. 2020;125(5):739-49.
