# Supplementary material for: Closed–Loop ventilation using sidestream versus mainstream capnography for automated adjustments of minute ventilation—A randomized clinical trial in cardiac surgery patients
Source: PLoS One. 2023 Aug 23;18(8):e0289412. doi: 10.1371/journal.pone.0289412 (PMC10446221; doi:10.1371/journal.pone.0289412)
Supplement: S1 Table — (DOCX) [file pone.0289412.s005.docx]

| **eTable 1 – Per protocol analysis of endpoints** | | | | | |
| --- | --- | --- | --- | --- | --- |
|  | **Sidestream**  **(N = 36)** | **Mainstream**  **(N = 37)** | **Mean Ratio**  **(95%–CI)** | **Absolute Mean Difference (95%–CI)** | ***P*** |
| Proportion of breaths in optimal zone |  | |  |  | 0.094* |
| Median [IQR] | 0.81 [0.51 – 0.91] | 0.85 [0.67 – 0.92] | 0.88 (0.78 to ∞) |  |  |
| Mean ± standard deviation | 0.69 ± 0.26 | 0.79 ± 0.17 |  |  |  |
| Proportion of breaths in acceptable zone |  | |  |  |  |
| Median | 0.13 [0.08 – 0.40] | 0.14 [0.08 – 0.29] | 1.36 (0.89 to 2.03) |  | 0.141 |
| Mean | 0.26 ± 0.23 | 0.19 ± 0.14 |  |  |  |
| Proportion of breaths in critical zone |  | |  |  |  |
| Median | 0.02 [0.01 – 0.03] | 0.01 [0.00 – 0.03] | 1.85 (0.71 to 5.01) |  | 0.169 |
| Mean | 0.05 ± 0.08 | 0.03 ± 0.04 |  |  |  |
| Proportion of time spent in optimal zone |  | |  |  |  |
| Median | 0.86 [0.51 – 0.91] | 0.85 [0.69 – 0.92] |  |  |  |
| Mean | 0.71 ± 0.24 | 0.79 ± 0.16 |  | -0.08 (-0.18 to 0.01) | 0.088 |
| Proportion of time spent in acceptable zone |  | |  |  |  |
| Median | 0.13 [0.08 – 0.40] | 0.14 [0.08 – 0.29] |  |  |  |
| Mean | 0.25 ± 0.22 | 0.19 ± .0.14 |  | 0.06 (-0.03 to 0.15) | 0.160 |
| Proportion of time spent in critical zone |  | |  |  |  |
| Median | 0.02 [0.00 – 0.03] | 0.01 [0.00 – 0.03] |  |  |  |
| Mean | 0.05 ± 0.09 | 0.03 ± 0.04 |  | 0.02 (-0.01 to 0.06) | 0.226 |
| Proportion of breaths with SpO_2_ < 85% |  | |  |  |  |
| Median | 0.00 [0.00 – 0.00] | 0.00 [0.00 – 0.00] | 2.51 (0.76 to 11.00) |  | 0.103 |
| Mean | 0.00 ± 0.01 | 0.00 ± 0.00 |  |  |  |
| ^*^*P* for non–inferiority.  Abbreviations: CI, confidence interval; SpO_2_, peripheral oxygen saturation. | | | | | |
