# Supplementary figures and images for: Closed–Loop ventilation using sidestream versus mainstream capnography for automated adjustments of minute ventilation—A randomized clinical trial in cardiac surgery patients
Source: PLoS One. 2023 Aug 23;18(8):e0289412. doi: 10.1371/journal.pone.0289412 (PMC10446221; doi:10.1371/journal.pone.0289412)

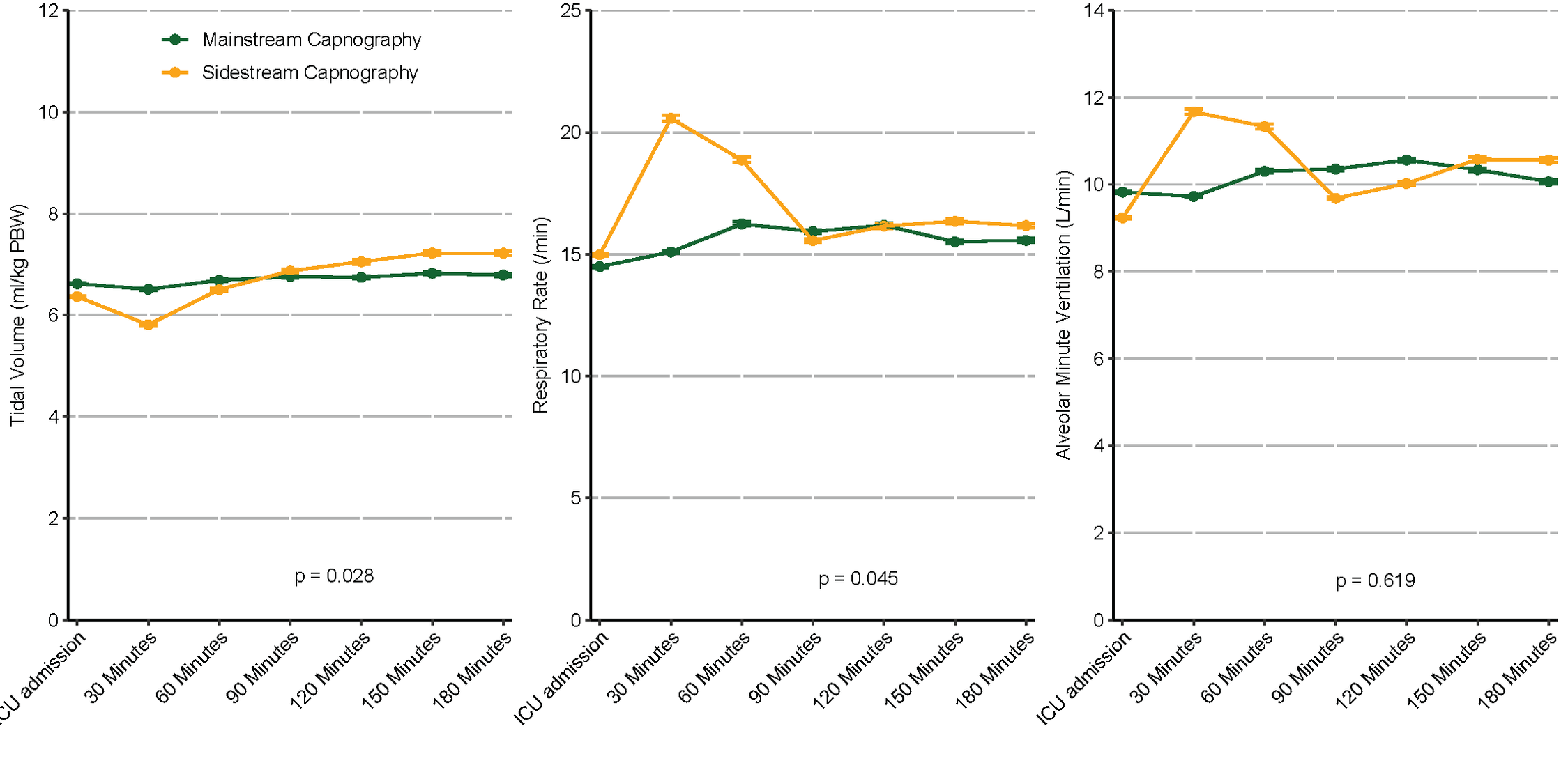

Supplement: S1 Fig — (TIF) [file pone.0289412.s006.tif]

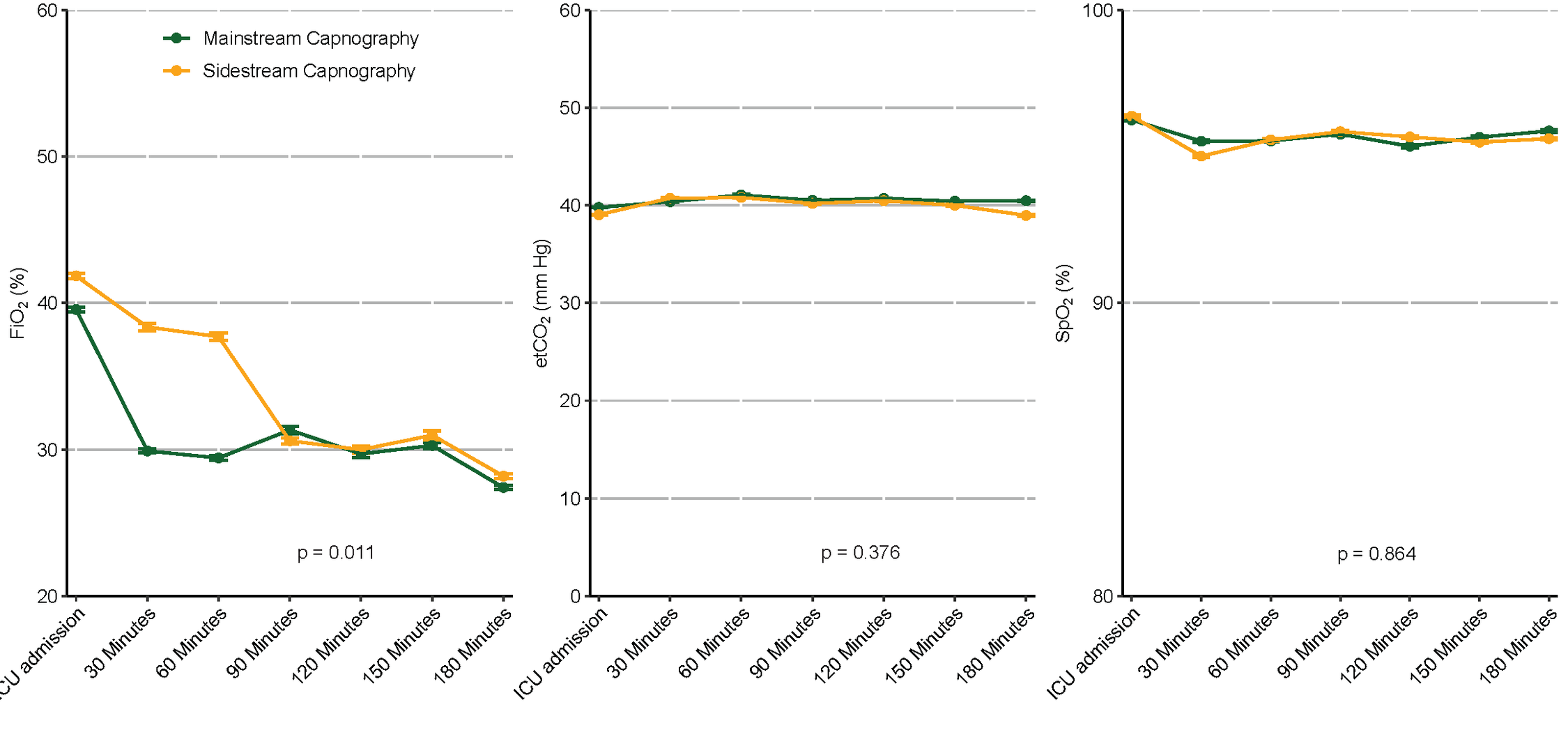

Supplement: S2 Fig — (TIF) [file pone.0289412.s007.tif]

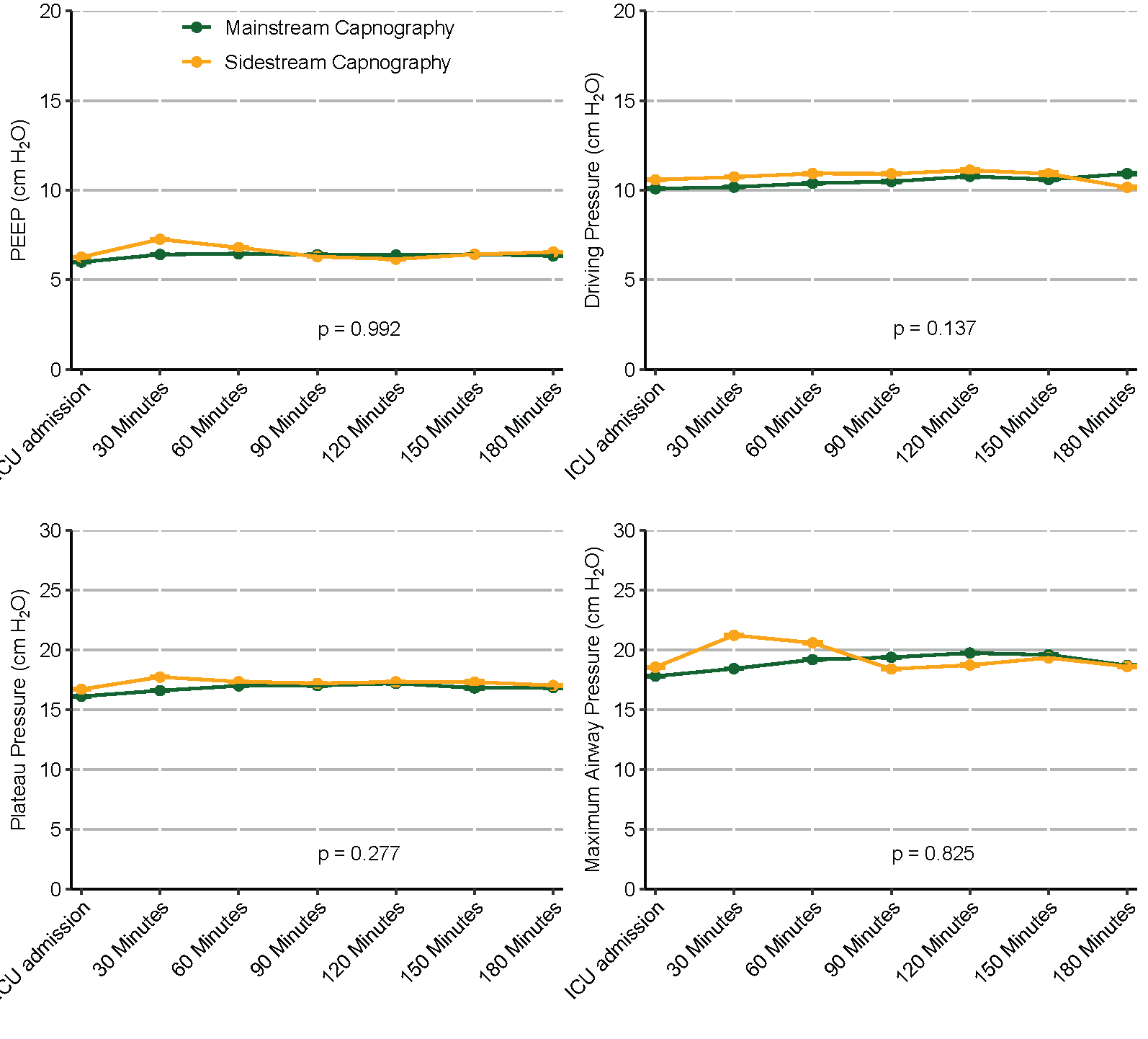

Supplement: S3 Fig — (TIF) [file pone.0289412.s008.tif]

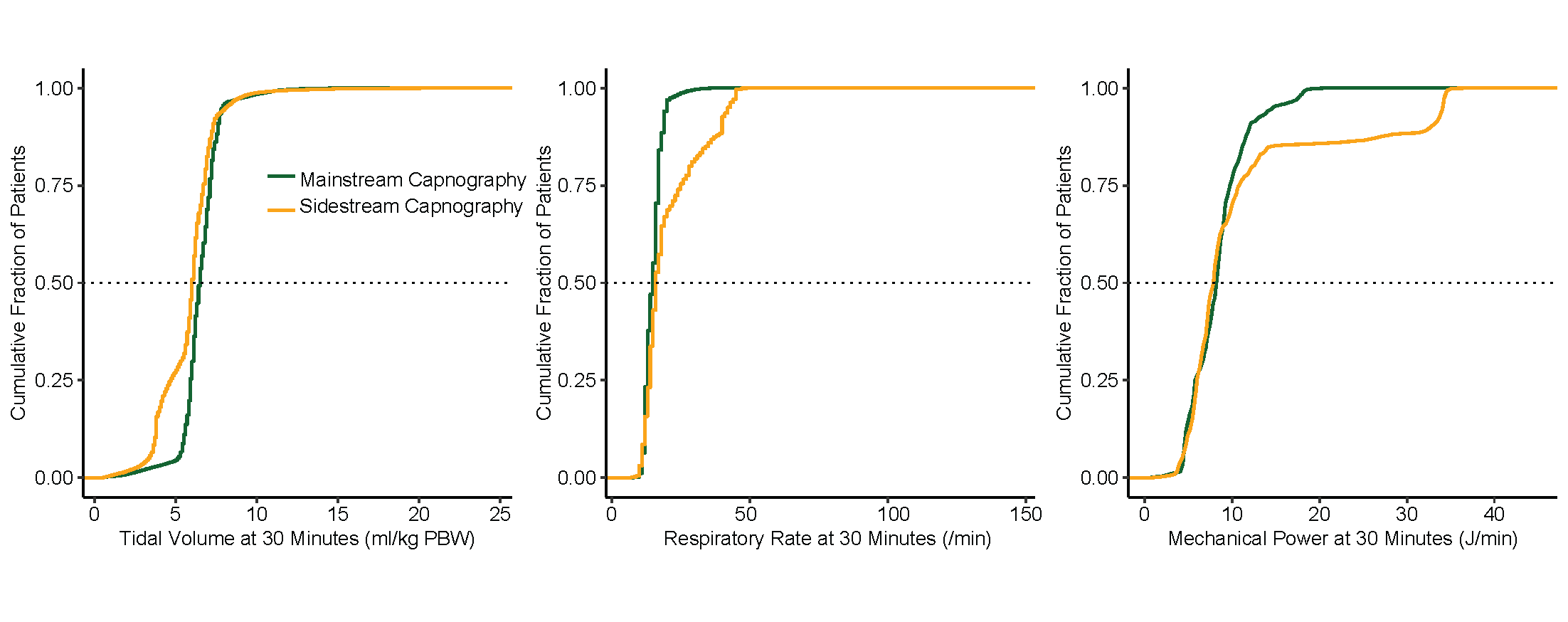

Supplement: S4 Fig — (TIF) [file pone.0289412.s009.tif]

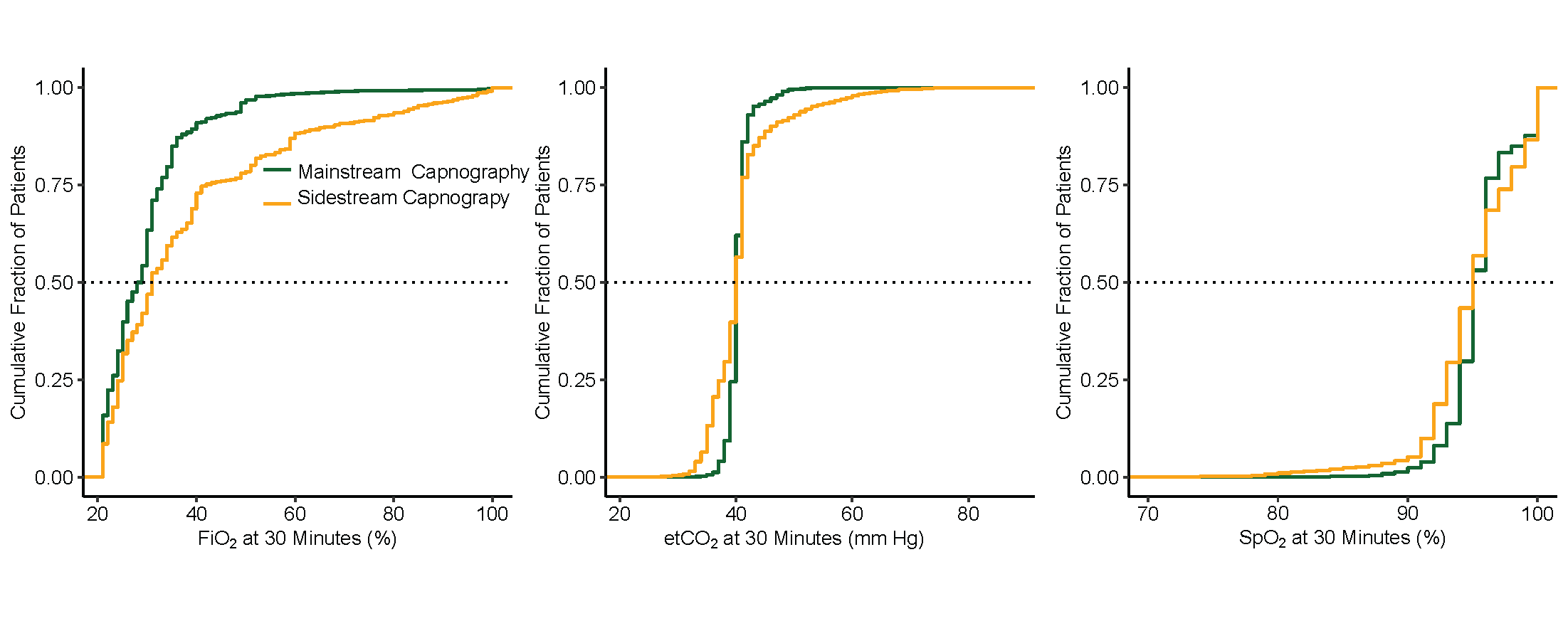

Supplement: S5 Fig — (TIF) [file pone.0289412.s010.tif]

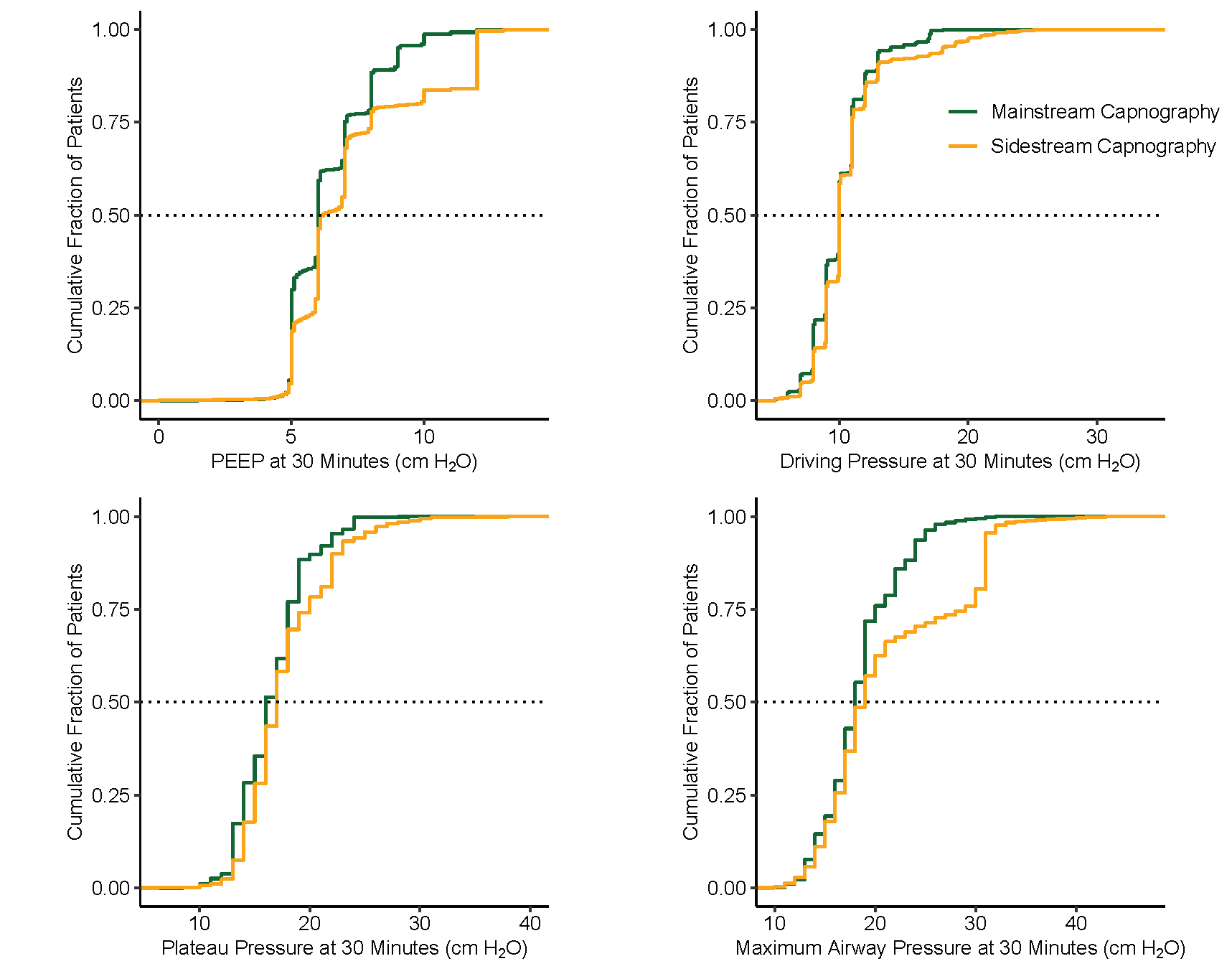

Supplement: S6 Fig — (TIF) [file pone.0289412.s011.tif]

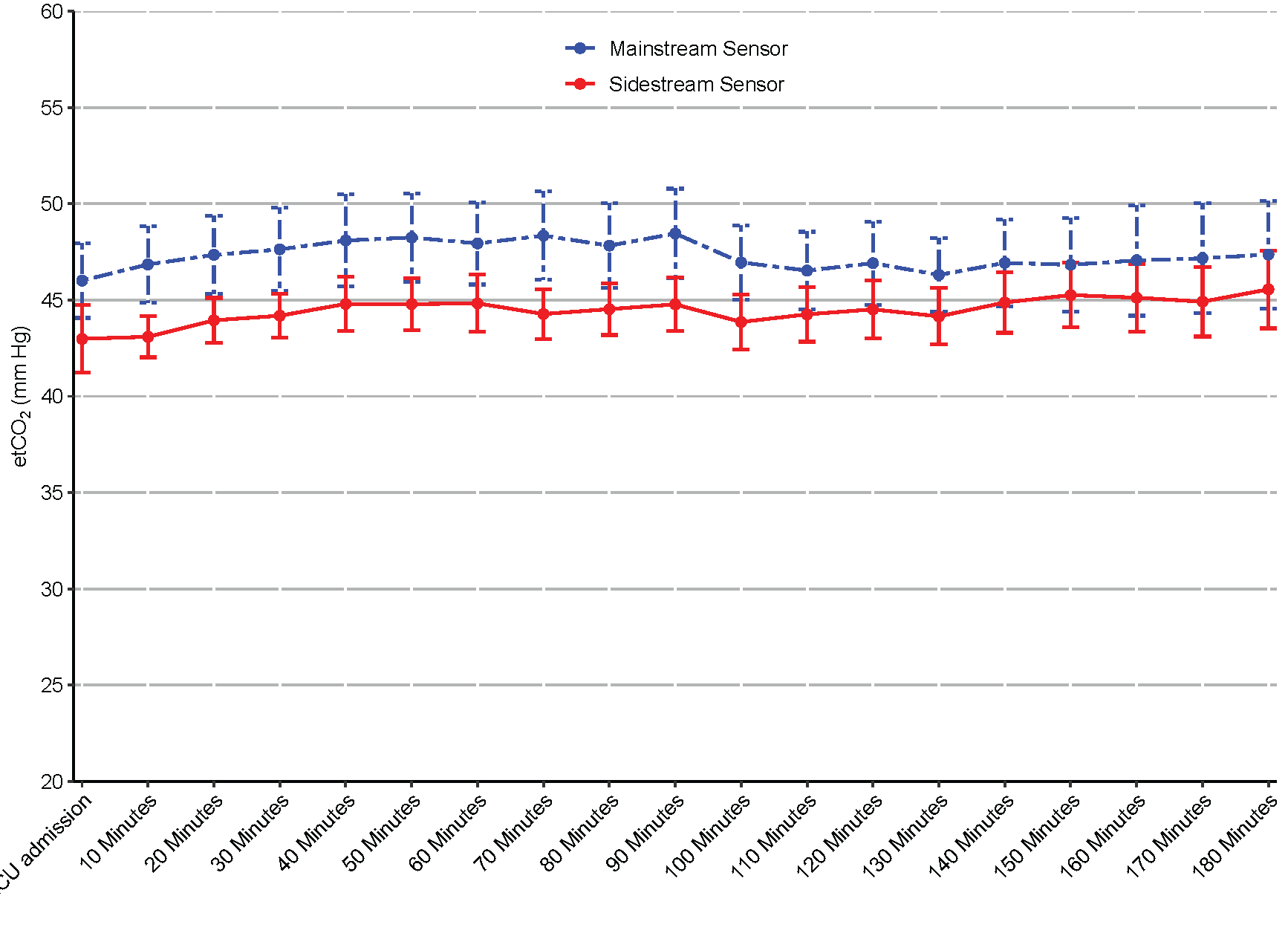

Supplement: S7 Fig — (TIF) [file pone.0289412.s012.tif]

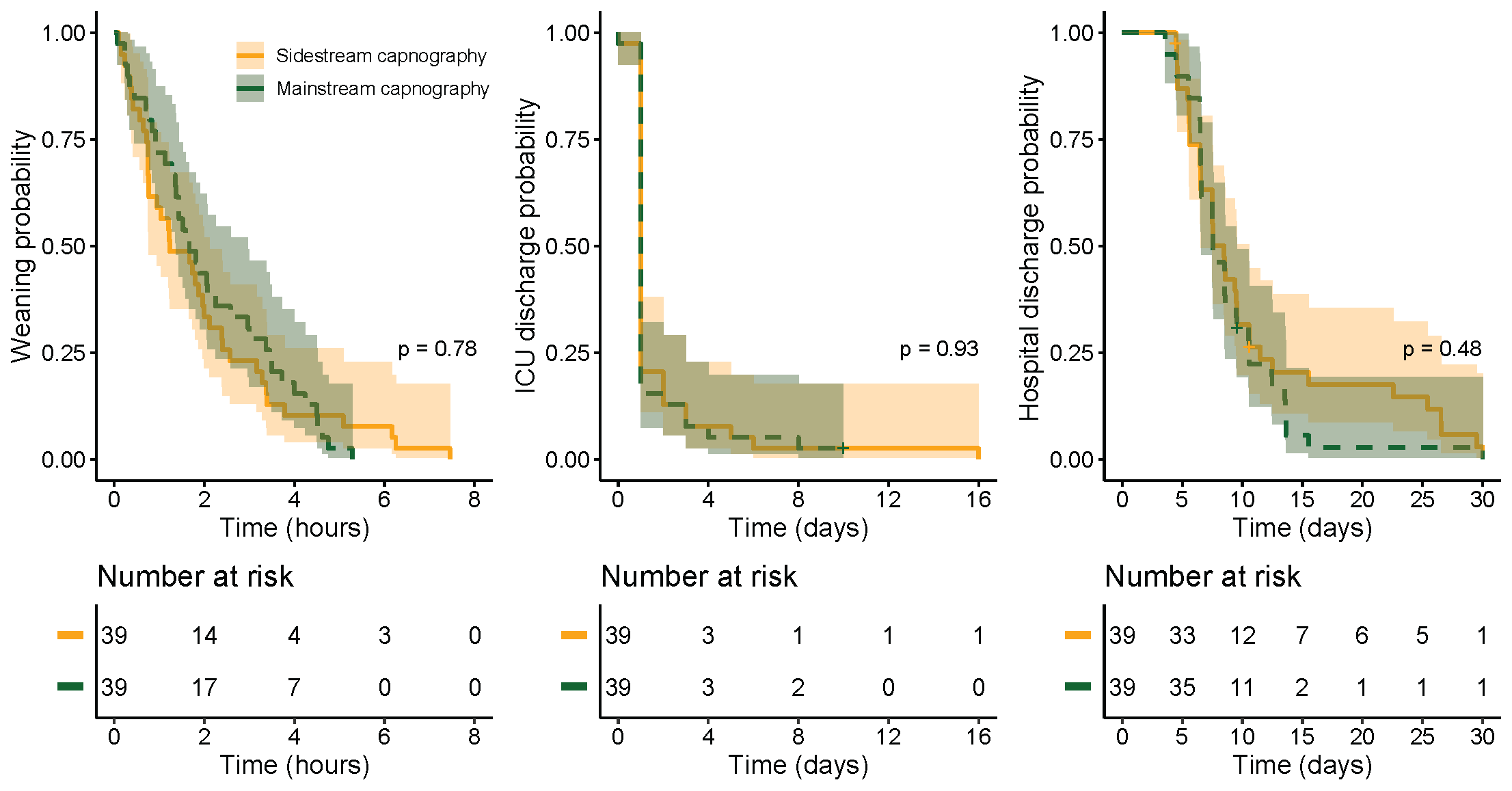

Supplement: S8 Fig — (TIF) [file pone.0289412.s013.tif]

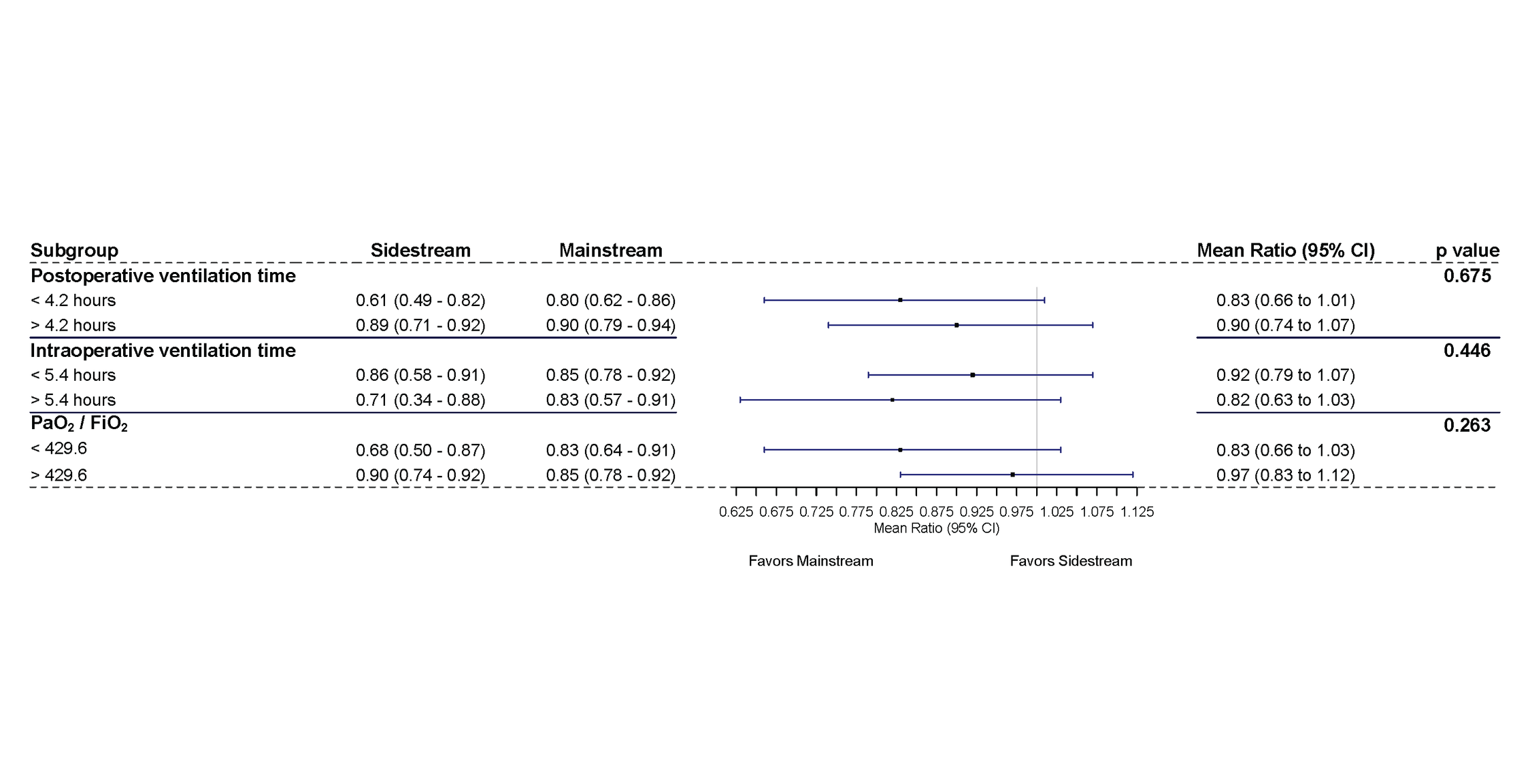

Supplement: S9 Fig — (TIF) [file pone.0289412.s014.tif]
